# Effect of twice daily inhaled albuterol on cardiopulmonary exercise outcomes, dynamic hyperinflation, and symptoms in secondhand tobacco-exposed persons with preserved spirometry and air trapping: a randomized controlled trial

**DOI:** 10.1186/s12890-023-02808-7

**Published:** 2024-01-20

**Authors:** Siyang Zeng, Melissa Nishihama, Lemlem Weldemichael, Helen Lozier, Warren M. Gold, Mehrdad Arjomandi

**Affiliations:** 1 Department of Biomedical Informatics and Medical Education, University of Washington, Seattle, WA USA; 2 Pulmonary and Critical Care Section, San Francisco Veterans Affairs Health Care System, Building 203, Room 3A-128, Mailstop 111-D, 4150 Clement Street, San Francisco, CA 94121 USA; 3 Department of Medicine, Division of Pulmonary and Critical Care Medicine, University of California, San Francisco, CA USA; 4 Carver College of Medicine, University of Iowa, Iowa City, USA; 5 Department of Medicine, Division of Occupational, Environmental, and Climate Medicine, University of California, San Francisco, CA USA

**Keywords:** Secondhand tobacco smoke, Air trapping, Spirometric obstruction, Tobacco-exposed person, Pre-COPD, Exercise capacity, Bronchodilation

## Abstract

**Background:**

In tobacco-exposed persons with preserved spirometry (active smoking or secondhand smoke [SHS] exposure), air trapping can identify a subset with worse symptoms and exercise capacity. The physiologic nature of air trapping in the absence of spirometric airflow obstruction remains unclear. The aim of this study was to examine the underlying pathophysiology of air trapping in the context of preserved spirometry and to determine the utility of bronchodilators in SHS tobacco-exposed persons with preserved spirometry and air trapping.

**Methods:**

We performed a double-blinded placebo-controlled crossover randomized clinical trial in nonsmoking individuals at risk for COPD due to exposure to occupational SHS who had preserved spirometry and air trapping defined as either a residual volume-to-total lung capacity ratio (RV/TLC) > 0.35 or presence of expiratory flow limitation (EFL, overlap of tidal breathing on maximum expiratory flow-volume loop) on spirometry at rest or during cardiopulmonary exercise testing (CPET). Those with asthma or obesity were excluded. Participants underwent CPET at baseline and after 4-week trials of twice daily inhalation of 180 mcg of albuterol or placebo separated by a 2-week washout period. The primary outcome was peak oxygen consumption (VO_2_) on CPET. Data was analyzed by both intention-to-treat and per-protocol based on adherence to treatment prescribed.

**Results:**

Overall, 42 participants completed the entire study (66 ± 8 years old, 91% female; forced expiratory volume in 1 s [FEV_1_] = 103 ± 16% predicted; FEV_1_ to forced vital capacity [FVC] ratio = 0.75 ± 0.05; RV/TLC = 0.39 ± 0.07; 85.7% with EFL). Adherence was high with 87% and 93% of prescribed doses taken in the treatment and placebo arms of the study, respectively (*P* = 0.349 for comparison between the two arms). There was no significant improvement in the primary or secondary outcomes by intention-to-treat or per-protocol analysis. In per-protocol subgroup analysis of those with RV/TLC > 0.35 and ≥ 90% adherence (*n* = 27), albuterol caused an improvement in peak VO_2_ (parameter estimate [95% confidence interval] = 0.108 [0.014, 0.202]; *P* = 0.037), tidal volume, minute ventilation, dynamic hyperinflation, and oxygen-pulse (all *P* < 0.05), but no change in symptoms or physical activity.

**Conclusions:**

Albuterol may improve exercise capacity in the subgroup of SHS tobacco-exposed persons with preserved spirometry and substantial air trapping. These findings suggest that air trapping in pre-COPD may be related to small airway disease that is not considered significant by spirometric indices of airflow obstruction.

**Supplementary Information:**

The online version contains supplementary material available at 10.1186/s12890-023-02808-7.

## Key messages

What is already known on this topic?

Many people who have been exposed to tobacco smoke (direct or indirect) but have preserved spirometry show abnormal lung volumes suggestive of presence of air trapping. The physiologic nature of air trapping in the absence of spirometric airflow obstruction remains unclear.

What this study adds?

Albuterol may improve ventilation and exercise capacity in secondhand tobacco-exposed persons with preserved spirometry and lung volumes suggestive of air trapping.

How this study might affect research, practice, or policy?

Air trapping in pre-COPD may be related to small airway disease that is not considered significant by spirometric indices of airflow obstruction. Stratification of the tobacco-exposed persons with preserved spirometry by lung volumes and air trapping may help in identifying a subset who do benefit from the use of bronchodilators.

**Supplementary Information:**

The online version contains supplementary material available at 10.1186/s12890-023-02808-7.

## Background

Chronic exposure to secondhand tobacco smoke (SHS) is a risk factor for development of chronic obstructive pulmonary disease (COPD) [1–3]. Lung function studies in people with history of long-term occupational exposure to SHS have shown the majority of those exposed to have preserved spirometry but also to have a remarkably wide distribution of lung volume indices that represent air trapping, specifically the ratio of residual volume or functional residual capacity to total lung capacity (RV/TLC or FRC/TLC) [4, 5]. Wide ranges of RV/TLC and FRC/TLC distributions have also been reported among people with history of direct smoking and preserved spirometry [6–8]. The significance of these observations continues to be a topic of investigation, though several recent studies have shown that in these unobstructed tobacco smoke-exposed people, lung volumes that represent air trapping (elevated RV/TLC and FRC/TLC) can identify a subset with worse symptoms and lower exercise capacity who, at least in the case of those with history of active smoking, will progress to develop overt airflow obstruction and spirometric COPD [5–7].

The physiologic nature of air trapping in the absence of spirometric airflow obstruction remains unclear, and a question remains on whether elevated RV/TLC or FRC/TLC is a consequence of an airway obstructive process that has not yet resulted in spirometric airflow obstruction by formal criteria, or the result of other yet unexplained lung parenchymal changes that cause an elevated RV/TLC and FRC/TLC without airway obstructive disease. A possible approach to investigate this question is to determine whether air trapping and its associated diminished exercise capacity and worse respiratory symptoms improve with the use of bronchodilators in this tobacco-exposed population with air trapping but preserved spirometry, based on the presumption that the main effect of bronchodilators is through its relief of airflow obstruction at the level of airways.

The main goal of this study was to understand the nature of air trapping in the setting of no spirometric obstruction in people at risk for COPD (pre-COPD) by investigating whether administration of bronchodilators in that setting could improve the adverse outcomes associated with air trapping including exercise tolerance. To investigate this, a clinical trial approach was taken, not to necessarily prove a recommendation for routine use of bronchodilators in pre-COPD, but rather to determine whether bronchodilation improves air trapping and its outcomes in that setting. We hypothesized that the exercise capacity and respiratory symptoms associated with air trapping in the absence of spirometric airflow obstruction (preserved spirometry) improve with administration of bronchodilators. To examine this hypothesis, we performed a clinical trial investigating the effectiveness of albuterol, a selective β2-adrenergic receptor agonist that relaxes airway smooth muscle and causes bronchodilation, in nonsmoking individuals at risk for COPD due to occupational exposure to SHS with preserved spirometry and air trapping, and assessed whether administration of albuterol improves their peak oxygen consumption (VO_2_) and other performance indices on cardiopulmonary exercise testing (CPET) as well as their level of symptoms, quality of life, and daily physical activity.

## Methods

### Study design

**Fig. 1:**
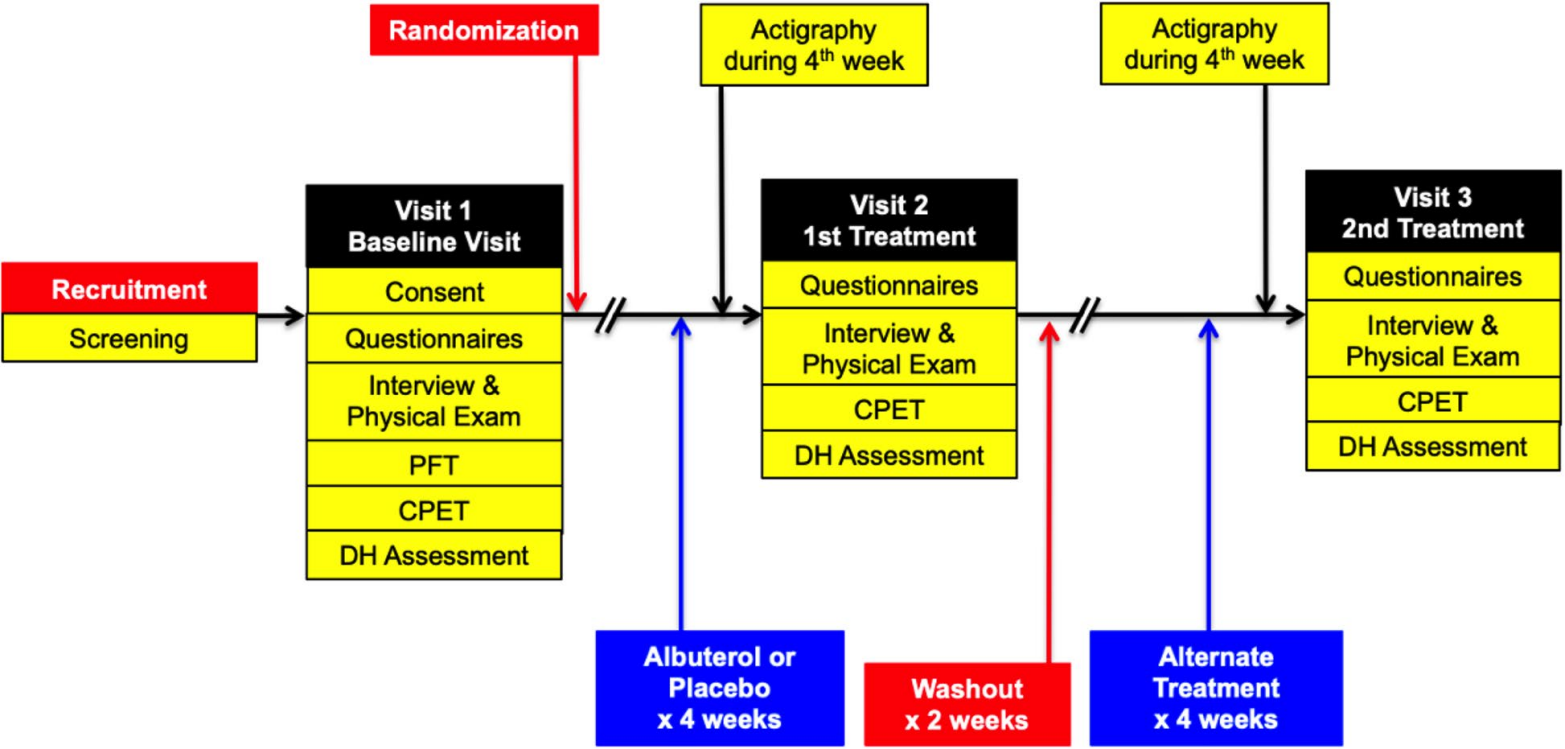
Study design. Abbreviations: PFT: pulmonary function test; CPET: cardiopulmonary exercise testing; DH: dynamic hyperinflation

This was a single-center, double-blinded, randomized, crossover, placebo-controlled trial investigating the effectiveness of albuterol in improving exercise capacity and respiratory symptoms in nonsmoking SHS tobacco-exposed individuals at risk for COPD due to occupational exposure to SHS with preserved spirometry and physiologic evidence of air trapping. Air trapping was defined as either (1) an absolute RV/TLC value > 0.35 on plethysmography or (2) presence of expiratory flow limitation (EFL) as defined by the presence of graphic overlap of tidal breathing on maximum expiratory flow-volume loop on spirometry at rest or during maximum effort exercise testing, regardless of their RV/TLC value. Potential participants underwent baseline characterization with questionnaire administration, pulmonary function testing (PFT), and CPET to determine eligibility. Eligible participants were then randomized to take twice daily inhalation of either albuterol (2 inhalations; 180 mcg) or placebo for 4 weeks before coming back in for a repeat evaluation. The assigned inhaler was taken up to and on the morning of evaluation. The dose on the morning of evaluation was a supervised administration about 30 to 60 min before the exercise testing. The participants subsequently underwent a washout period of at least 2 weeks duration, after which they were crossover assigned to take the alternate treatment (albuterol or placebo) for 4 weeks followed by repeat evaluation. Participants also wore an activity monitor during the last week of each treatment period (Fig. 1).

The primary outcome of the study was peak VO_2_ on CPET. The secondary outcomes included improvement in other CPET indices including EFL and dynamic hyperinflation as well as symptoms, quality of life, and daily level of physical activity. Data was analyzed by both intention-to-treat and per-protocol approaches. The per-protocol approach was based on adherence defined as the number of albuterol or placebo inhalations that the participants actually took as a fraction of the total number of inhalations that they were instructed to take.

### Trial regulatory matters

The University of California San Francisco (UCSF) Institutional Review Board (IRB) and the San Francisco Veterans Affairs Health Care System (SFVAHCS) Committee on Research and Development approved the study protocols. Written IRB-approved informed consent and Health Insurance Portability and Accountability Act (HIPAA) were obtained from all study participants. All participants received monetary compensation for their participation in the study. The study was registered with the United States (U.S.) National Library of Medicine (Secondhand Smoke Respiratory Health Study; ClinicalTrials.gov identifier NCT02797275; date first posted in the trial registration 13/06/2016 [June 13, 2016]).

### Study population

The study population consisted of past or current U.S. commercial airline flight crewmembers who had previously participated in an observational study of the potential adverse health effects of the cabin environment including health effects of exposure to occupational SHS in the aircraft cabin [5, 9, 10]. Crewmembers were eligible to participate in the study if they were between 40 and 80 years of age, nonsmokers, had worked onboard of planes for at least one year before enactment of tobacco smoking ban in aircraft, and had preserved spirometry (defined as a ratio of forced expiratory volume in 1 s to forced vital capacity (FEV_1_/FVC) ≥ 0.70 and greater than lower limit of normal value (LLN). The participants also had to have evidence of air trapping as determined by either (1) an absolute RV/TLC ratio of > 0.35 by plethysmography (this threshold was used based on previous work by our group showing this RV/TLC level to be a statistically optimal cutoff point for RV/TLC association with presence of exercise limitation and symptoms) [5]; or (2) EFL defined as the presence of overlap of tidal breathing on maximum expiratory flow-volume loop graph on spirometry at rest or during their baseline maximum effort exercise testing, regardless of their RV/TLC value (EFL with exercise is suggestive of progressive air trapping and dynamic hyperinflation, which correlate better with symptoms and functional capacity than spirometric indices) [11, 12].

Those with history of asthma, COPD, interstitial lung diseases, active cardiovascular disease, uncontrolled hypertension, auto-immune diseases, or other known conditions affecting lung function (e.g., history of radiation therapy to chest in the setting of history of breast cancer), obesity (defined as body mass index or BMI > 30 kg/m^2^), or history of cannabis or other recreational drug use were excluded.

### Randomization

Randomization of the participants occurred between June 6, 2016 and February 27, 2020. Randomization for each individual participant to receive albuterol or placebo as their first inhaler was done using a random number generator. A random number between 0 and 1 was generated for each eligible participant. Participants with a random number ≥ 0.5 received albuterol as their first inhaler while participants with a random number < 0.5 received placebo as their first inhaler. The randomization was blinded from the participants and from the study team. A staff member, who was otherwise not involved in the study, performed the randomization and then delivered the assigned inhalers to the participants.

### Study medication

Albuterol is a selective β2-adrenergic receptor agonist, inhalation of which causes relaxation of airway smooth muscle and bronchodilation [13, 14]. Albuterol has a terminal half-life of 3 to 8 h and was considered to be a “long-acting” agent in 1980s [14], but with the advent of other agents with much longer duration of action (long-acting and ultra-long-acting agents) [15–17], albuterol is now considered to be a “short-acting” β2-agonist. Both short-, long-, and ultra-long-acting β2-agonists can interact with adrenergic receptors on other cells and organ systems and cause potentially unwanted effects including plasma electrolytes derangement, cardiovascular effects such as tachycardia, and skeletal muscle tremor [17, 18], although these effects are most common with systemic (oral or parenteral) rather than inhalational routes of administration. In lungs, inhalation of albuterol and other β2-agonists are thought to mainly affect airway smooth muscle cells and have a bronchodilatory effect, although effects on other lung cells (such as airway and alveolar epithelial cells as well as airway immune cells) have also been documented [19–24].

Administration of albuterol for the study was performed using ProAir RespiClick metered inhalation powder (albuterol sulfate 90 mcg per inhalation) (Teva Respiratory, LLC., Parsippany, NJ, USA), as the intervention drug and the Demo version of RespiClick (obtained from ProAir manufacturer) that contained no active medication, as the placebo. The participants were instructed to take two inhalations of either albuterol (2 inhalations; 180 mcg) or placebo twice daily for 4 weeks before coming back in for a repeat evaluation. The assigned inhaler was taken up to and on the morning of evaluation. The dose on the morning of evaluation was a supervised administration about 30 to 60 min before the exercise testing. The 4-week duration of albuterol administration was chosen based on the previous literature showing that despite normalization of any spirometric airflow obstruction, reversal of air trapping and hyperinflation may require weeks of therapy to resolve [25].

The inhalers were provided to participants after randomization in their first visit by a staff member who was otherwise not involved with the study. The same staff member reviewed the correct use of the inhalers with the participants. To avoid any possible adrenergic adverse effects at night, the participants were instructed to use two inhalations of the ProAir RespiClick in the morning upon awakening and two more inhalations about six hours later in the early afternoon, with no further usage after that throughout the evening and night. The participants were contacted by the study staff at one week and three weeks after the initiation of medication to assess their usage and any potential adverse effects. The participants were also asked to use the study medication on the day they returned to the laboratory for their follow-up visits (V2 and V3).

During the participants’ follow-up visits (V2 and V3), the same staff member who dispensed the inhaler to the participants collected the inhalers from them, and noted and recorded the number of inhalations that was used from the built-in use counter for later calculation of the rate of adherence to the protocol. The data on adherence to inhaler use was unblinded and calculated after the end of the study on September 9, 2022.

### SHS exposure characterization

Details of SHS exposure characterization and quantification are available in the Supplemental Appendix and have been described previously [4].

### Pulmonary function and cardiopulmonary exercise testing

Participants performed physician-supervised, symptom-limited, progressively increasing stepwise maximal exercise tests in the seated position on an electromagnetically braked, upright cycle ergometer with continuous monitoring of their heart rate (HR), blood pressure (BP), electrocardiogram (ECG), and breath-by-breath gas exchange. Approximately 45 min after completing their maximum effort exercise testing, the participants underwent a dynamic hyperinflation exercise testing protocol. This protocol included tidal volume (V_T_), inspiratory capacity (IC), and maximal expiratory flow (MEF) measurements in seated position at increasing work rates corresponding to 20%, 40%, 60%, and 80% of the peak VO_2_ they attained during the maximum effort exercise testing, as described by O’Donnell et al [26, 27]. Details of pulmonary function and maximum effort cardiopulmonary function testing are available in the Supplemental Appendix and have been described previously [4, 5].

### Physical activity monitoring using actigraphy

Physical activity was monitored using a triaxial accelerometer-based activity monitor (ActiGraph GT3X; Actigraph Corporations, Pensacola, FL). Technical details of the device, data processing, and data analysis are available in the Supplemental Appendix and have been described previously [28].

### Respiratory symptom assessment

Patient-reported respiratory symptoms, physical activity, and quality of life assessments were conducted using the COPD Assessment Test (CAT) [29], modified Medical Research Council (mMRC) Dyspnea Scale [30], the Short Form 12-Item Health Survey (SF12) [31], International Physical Activity Questionnaire (IPAQ) [32], Airway Questionnaire 20 (AQ20) [33], and a self-reported questionnaire (UCSF Flight Attendant Medical Research Institute (FAMRI) SHS Questionnaire) that elicited symptoms of dyspnea, cough, and participants’ perception of a decreased level of exertion compared to peers over the year preceding enrollment [34], further details of which are available in the Supplemental Appendix.

### Power and sample size calculations

The a priori power and sample size calculations were performed as described below. Previous studies have reported high level of correlation (coefficient of correlation ≥ 0.90 and coefficient of variation < 7%) between repeated measurements of peak VO_2 _in maximum effort exercise testing among healthy as well as diseased populations [35–38]. For the purpose of this study with a repeated measure design, we used a presumed correlation coefficient of 0.90. Moreover, previous CPET data from a relatively large cohort of the SHS-exposed flight attendants with preserved spirometry and air trapping have documented peak VO_2 _measures (mean ± standard deviation) of 1.496 ± 0.429 L/min [5]. Given the above, and assuming that some 80% of the participants would have a small airway disease process that is responsive to administration of bronchodilator, a sample size of 103 was determined to provide a power of 80% to detect 10% change in peak VO_2_ (or 0.15 L/min). In the end, we planned to recruit a total of 100 participants, although because of various impediments including research holds related to COVID-19 pandemic, the targeted sample size was not achieved.

### Data analysis

Distributions of participants’ characteristics, pulmonary function, cardiopulmonary exercise, and SHS exposure quantification variables were examined. Rate of change in cardiopulmonary exercise measures during the exercise with respect to the workload were approximated by estimating the slopes from linear regression modeling of those measures over workload at each stage. Peak cardiopulmonary exercise variables were estimated using the last 30-s average values obtained during the highest stage of the exercise test as described in Supplemental Appendix. Cumulative work achieved throughout the exercise (Work_Total_), or the area under the curve of workload in Watts vs. time in minutes, was computed as the sum of the product of watts completed and time spent at each stage in the unit of Watts-Minute. Changes in the outcomes from baseline were calculated by subtracting the subsequent visits (V2 or V3) outcome values from those of baseline visit (V1).

#### Intention-to-treat analysis

The primary intention-to-treat analysis was performed as described below. The effect of albuterol on the outcome variables was examined with a repeated measure design using mixed-effect linear regression modeling with the random subject effect and fixed effect variables including age, sex, height, weight, and the corresponding baseline measure of the outcome variable. Participants who completed only one arm of the study were included in this analysis (*intention-to-treat*). The resulted coefficients representing the adjusted difference between the outcome variables measured in the albuterol visit and the placebo visit were reported.

The changes after administration of albuterol were also compared with those after administration of placebo using paired t-test. Changes that were significantly different between taking albuterol and taking placebo were reported.

#### Per-protocol and subgroup analyses

The a priori per-protocol analysis was performed as described below. To assess the effect of albuterol under different levels of adherence, per-protocol analyses using the same methodology as described above for intention-to-treat analysis were performed based on thresholds of adherence. Thresholds of adherence of 70%, 80%, and 90% were applied to each arm of the study. We then analyzed the subgroups of participants whose adherence data was equal or above the thresholds.

To assess whether the treatment was effective in the subgroup who had definitive air trapping (excluding those with EFL who did not have an RV/TLC > 0.35), *post-hoc* subgroup analyses were also performed based on the thresholds of adherence and RV/TLC. To evaluate any potential interaction between other lung function indices that represent small airway disease or air trapping and the effect of albuterol treatment on the study outcomes, we performed additional regression analyses with inclusion of these variables (forced expiratory flow [FEF] at 25% to 75% of FVC [FEF_25-75_], FEF at 75% of FVC [FEF_75_], ratio of alveolar volume [VA] to TLC) as an independent variable as well as an interaction term within the models.

For each analysis, the total number of participants included for that analysis were reported along with the results from the regression modeling and paired t-test. R software (version 4.2.2; R Foundation for Statistical Computing) was used for data management and statistical analysis. Figures were generated by GraphPad (Prism version 9.0).

## Results

### Participant characteristics

**Fig. 2:**
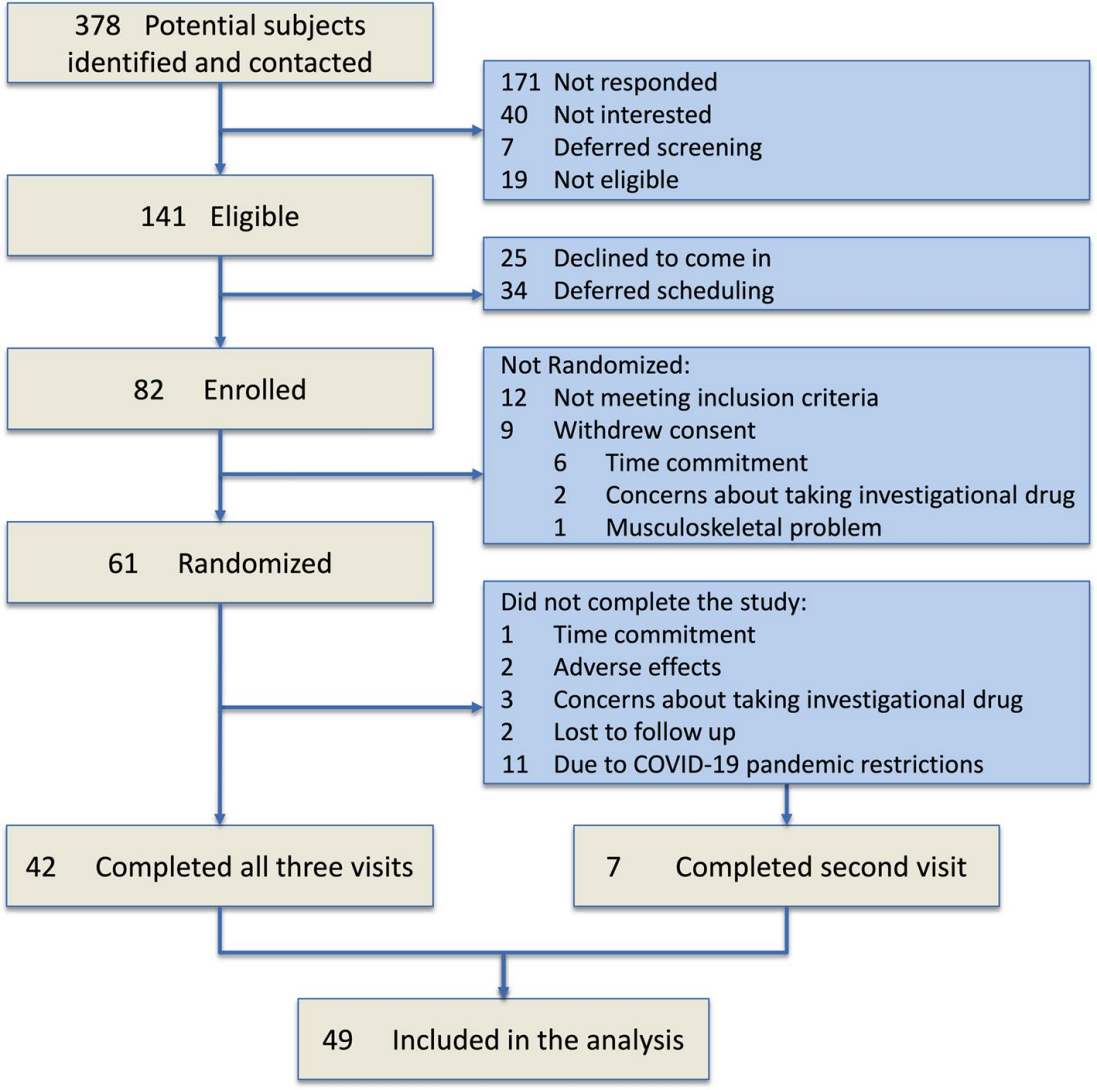
Participants flow through the study

Between June 6, 2016 and February 27, 2020, 378 potential participants were identified and contacted. From those who responded and were interested, 141 met the initial eligibility criteria, and 82 ended up being enrolled in the study (Fig. 2). From the 61 participants randomized, six withdrew, two lost to follow-up, and eleven were not able to complete the study due to institutional holds on clinical research during the COVD-19 pandemic. Overall, 42 completed the entire study. In addition, seven participants who completed the second study visit were also included in the analysis. The characteristics of the 49 participants with available data for analysis are shown in Table 1. From the 49 participants, 27 had air trapping by RV/TLC > 0.35 at baseline and ≥ 90% adherence to inhaler use and were examined in a subgroup analysis. The characteristics of the 42 participants who completed the entire study and the 27 participants in the subgroup analysis are shown in Supplemental Table S1. The participants were predominantly women (43 out of 49 or 88%), 66 ± 8 years of age, and all nonsmokers with history of past exposure to SHS.

**Table 1:** Participant characteristics at baseline

Participant characteristics	Albuterol w/ V2 Placebo w/ V3 (*N* = 29)	Placebo w/ V2 Albuterol w/ V3 (*N* = 20)	Overall (*N* = 49)
**Demographics and anthropometrics**			
Age (years)	66.2 ± 8.9	66.3 ± 6.5	66.2 ± 8.0
Female sex [n (%)]	25 (86.2%)	18 (90.0%)	43 (87.8%)
Height (cm)	165.5 ± 7.8	167.9 ± 8.6	166.5 ± 8.1
Weight (kg)	65.3 ± 13.5	69.1 ± 12.0	66.9 ± 12.9
BMI (kg/m^2^)	23.7 ± 3.5	24.5 ± 3.9	24.0 ± 3.7
Hemoglobin (g/dL)	13.6 ± 0.4	13.5 ± 0.4	13.5 ± 0.4
**SHS Exposure**			
Ever Cabin SHS exposure [n (%)]	27 (93.1%)	14 (70.0%)	41 (83.7%)
Cabin SHS exposure among exposed (years)	16.1 ± 9.6	20.4 ± 7.5	17.6 ± 9.0
Any form of non-cabin SHS exposure [n (%)]	29 (100%)	20 (100%)	49 (100%)
Childhood home SHS exposure [n (%)]	18 (62.1%)	11 (55.0%)	29 (59.2%)
Adult home SHS exposure [n (%)]	12 (41.4%)	9 (45.0%)	21 (42.9%)
Non-airline occupational SHS exposure [n (%)]	24 (82.8%)	18 (90.0%)	42 (85.7%)
Other SHS Exposure [n (%)]	26 (89.7%)	17 (85.0%)	43 (87.8%)
**Symptoms**			
mMRC Dyspnea Scale ≥ 1 [n (%)]	5 (31.3%)	5 (38.5%)	10 (34.5%)
SF12			
Physical component score	37.9 ± 4.3	39.3 ± 5.7	38.5 ± 4.9
Mental component score	48.9 ± 3.8	48.9 ± 5.7	48.9 ± 4.6
IPAQ score			
High [n (%)]	25 (86.2%)	18 (90.0%)	43 (87.8%)
Moderate [n (%)]	4 (13.8%)	1 (5.0%)	5 (10.2%)
Low [n (%)]	0 (0%)	1 (5.0%)	1 (2.0%)
CAT	6.0 ± 5.6	7.4 ± 5.9	6.6 ± 5.7
**Pulmonary Function Tests**			
FEV_1_ (% predicted)	102 ± 18	105 ± 9	103 ± 16
FVC (% predicted)	106 ± 19	110 ± 8	107 ± 16
FEV_1_/FVC	0.75 ± 0.05	0.75 ± 0.04	0.75 ± 0.05
FEF_25-75_ (% predicted)	99 ± 33	96 ± 26	98 ± 30
FEF_75_ (% predicted)	127 ± 55	127 ± 59	127 ± 56
D_LCO_ adjusted for Hgb (% predicted)	82 ± 12	85 ± 13	84 ± 12
TLC (% predicted)	99 ± 12	103 ± 11	101 ± 11
RV (% predicted)	96 ± 15	100 ± 18	98 ± 16
RV/TLC	0.39 ± 0.07	0.38 ± 0.05	0.39 ± 0.07
RV/TLC (% predicted)	95 ± 15	92 ± 10	94 ± 13
FRC (% predicted)	94 ± 15	99 ± 20	96 ± 17
FRC/TLC	0.51 ± 0.08	0.51 ± 0.07	0.51 ± 0.07
FRC/TLC (% predicted)	92 ± 13	92 ± 13	92 ± 13
**Cardiopulmonary Testing Measurements**			
VO_2Peak_ (% predicted)	96 ± 22	104 ± 18	99 ± 21
VO_2Peak_/kg (% predicted)	82 ± 17	92 ± 24	86 ± 20
RER_Peak_ (% predicted)	103 ± 7	103 ± 8	103 ± 8
Watts_Peak_ (% predicted)	116 ± 28	114 ± 29	115 ± 28
Total duration (minute)	9.6 ± 1.9	10.2 ± 2.2	9.8 ± 2.0
Symptoms at peak exercise (Borg Scale 0 to 10)			
Shortness of Breath	5.43 ± 1.73	5.85 ± 2.03	5.60 ± 1.85
Effort	6.00 ± 1.91	6.70 ± 2.25	6.29 ± 2.06
Fatigue	5.50 ± 1.64	6.30 ± 2.13	5.83 ± 1.88
**Pulmonary Response**			
V_EPeak_ (% predicted)	56 ± 12	55 ± 13	56 ± 12
RR_Peak_ (% predicted)	63 ± 12	59 ± 15	61 ± 13
V_TPeak_ (% predicted)	94 ± 20	99 ± 18	96 ± 19
V_E_/VCO_2Peak_	36.2 ± 11.9	31.9 ± 4.4	34.4 ± 9.8
V_E_/VCO_2Peak_ (% predicted)	90 ± 30	80 ± 11	86 ± 24
VO_2_ at Anaerobic Threshold (VO_2AT_) (% predicted)	76 ± 23	89 ± 21	81 ± 23
**Cardiovascular Response**			
HR_Peak_ (% predicted)	93 ± 15	90 ± 12	92 ± 14
HR_Peak_ ≥ 90% predicted [n (%)]	18 (62.1%)	10 (50.0%)	28 (57.1%)
HR_Peak_ ≥ 80% predicted [n (%)]	25 (86.2%)	16 (80.0%)	41 (83.7%)
SBP_Rest_ (mmHg)	130 ± 17	125 ± 16	128 ± 17
SBP_Peak_ (mmHg)	191 ± 29	185 ± 29	189 ± 29
DBP_Rest_ (mmHg)	72 ± 10	74 ± 9	73 ± 9
DBP_Peak_ (mmHg)	85 ± 11	83 ± 11	84 ± 11
O_2_-Pulse_Peak_ (% predicted)	100 ± 29	114 ± 31	106 ± 30
SpO_2_ nadir (at peak exercise)	97.4 ± 1.5	97.2 ± 1.5	97.3 ± 1.5
**Dynamic Hyperinflation**			
VFL_Rest_ (L)	0.23 ± 0.24	0.16 ± 0.27	0.20 ± 0.25
VFL_80% effort_ (L)	0.60 ± 0.33	0.49 ± 0.40	0.55 ± 0.36
VFL_Slope_ (mL/watt)	3.93 ± 3.43	3.62 ± 3.38	3.80 ± 3.38
EFL_Rest_ (%)	21.9 ± 23.6	15.8 ± 26.4	19.5 ± 24.7
EFL_80% effort_ (%)	32.8 ± 16.4	24.9 ± 20.7	29.7 ± 18.4
EFL_Slope_ (%/watt)	0.10 ± 0.25	0.08 ± 0.21	0.09 ± 0.23
EFL at rest [n (%)]	16 (57.1%)	7 (35.0%)	23 (46.9%)
EFL at rest or during exercise [n (%)]	27 (93.1%)	16 (80.0%)	43 (87.8%)
EELV_Rest_	2.68 ± 0.47	3.04 ± 0.80	2.82 ± 0.64
EELV_80% effort_	2.68 ± 0.49	2.87 ± 0.72	2.76 ± 0.59
Slope of EELV across exercise stages (mL/watts)	0.06 ± 4.21	-1.78 ± 2.66	-0.69 ± 3.74
No. with increase in EELV slope [n (%)]	16 (55.2%)	5 (25.0%)	21 (42.9%)

*Footnote*: Demographics, secondhand smoke (SHS) exposure, symptoms, and lung function in participants with preserved spirometry that underwent exercise testing. Other SHS exposure was defined as non-aircraft cabin SHS exposure outside the work or home environment such as in recreational public places. Data are presented as mean ± standard deviation or number of participants with positive value for the variable (n) out of the total number of participants (N) and percentage of participants (%). Reference equations: percent predicted of normal values of spirometry, diffusing capacity, and lung volumes were calculated using Global Lung Function Initiative (GLI), Crapo, and Stock and Quanjer predicted formulas, respectively [39–42]. Percent predicted of normal values of cardiopulmonary outputs were calculated using Wassermann predicted formulas [39]. Available measures of the variables at peak exercise, at rest, and at anaerobic threshold were reported. Rate of change in the variables during the exercise testing were assessed by linear regression slope of the variables with respect to the workload

*Abbreviations*: *BMI* body mass index, *mMRC* modified medical research council, *SF12* Short Form 12-Item Health Survey, *IPAQ* International Physical Activity Questionnaire, *CAT* COPD Assessment Test, *FEV*_*1*_ forced expiratory volume in 1 s, *FVC* forced vital capacity, *FEF*_*25-75*_ maximum airflow at mid-lung volume, *FEF*_*75*_ maximum airflow at low-lung volume, *TLC* total lung capacity, *RV* residual volume, *FRC* functional residual capacity, *D*_*LCO*_ single-breath diffusing capacity of carbon monoxide, *Hgb* hemoglobin, *VO*_*2*_ oxygen uptake, *VO*_*2Peak.kg*_ peak oxygen uptake per kilogram of body weight, *Watts* work stage completed in watts, *VCO*_2_ carbon dioxide production, *V*_E_ minute ventilation value, *RER* respiratory exchange ratio (VCO_2_/VO_2_) at peak exercise, *RR* respiratory rate, *V*_*T*_ tidal volume, *HR* heart rate, *HRR* heart rate reserve, *SBP* systolic blood pressure, *DBP* diastolic blood pressure, *O*_*2*_*-Pulse* oxygen uptake per heartbeat, *SpO*_*2*_ oxygen saturation, *VFL* volume of the tidal breath that is flow limited on expiration, *EFL* expiratory flow limitation, *EELV* end-expiratory lung volume, Slope of EELV across exercise stages: estimate of regression coefficient of three EELV measurements at each of baseline (rest), 20%, 40%, 60%, and 80% of the load intensity (watts) of the peak exercise stage achieved

### Adherence and safety

Adherence to treatment protocol for ≥ 70%, ≥ 80%, and ≥ 90% of doses taken was 95 ± 7%, 96 ± 5%, and 98 ± 3%, respectively. Adherence in the treatment (albuterol) and control (placebo) arms were 87% and 93% of prescribed doses, respectively (*P* = 0.349 for comparison between the two arms). No serious adverse events were reported by the participants. Two participants stop their participation, one while in the treatment arm and another while in the placebo arm, due to non-serious adverse events, which were deemed unlikely to be related to treatment or placebo (knee pain and high blood pressure for the participant in the treatment arm and diarrhea for the participant in the placebo arm).

### Baseline lung function and exercise data

All participants had preserved spirometry as defined by FEV_1_ to FVC ratio ≥ 0.7 and greater than lower limit of predicted normal values (LLN) (Table 1). While the average diffusing capacity was within the normal limits, 12 of the 49 participants had diffusing capacities below LLN. Similarly, while the average FRC, RV, and TLC values were within the normal limits, two, one, and three of the 49 participants had FRC, RV, and TLC above the upper limit of normal values (ULN), respectively. Of note, 4 of the 12 participants with diffusing capacity < LLN had RV/TLC ≤ 0.35 but had EFL both at rest and during exercise.

The volume of oxygen consumption at peak exercise (VO_2_) was 1,335 ± 414 mL/min (99 ± 21% predicted). Thirteen participants (27%) had a VO_2Peak_ < 84% predicted, a presumed threshold for abnormal results. The peak VO_2_ was achieved at a peak workload of 113 ± 36 watts (115 ± 28% predicted) (Table 1) and over a period of 9.6 ± 1.8 min of loaded exercise.

None of the participants reported adverse effects during the CPET, including no chest pain, chest tightness, lightheadedness, or dizziness. Furthermore, no participants had any clinically significant ECG changes or arrhythmias, with the exception of occasional premature ventricular contractions that were not exercise dependent. The nadir of the ratio of oxy-hemoglobin to total hemoglobin (oxygen saturation or SpO_2_) at peak exercise was 97 ± 2% with all participants having an SpO_2_ of ≥ 95%. Thirty-four participants (72%) showed a hypertensive response to exercise. At peak exercise, the heart rate and oxygen-pulse (O_2_-Pulse) were 141 ± 22 beat/min (92 ± 14% predicted) and 9.7 ± 3.8 mL/beat (106 ± 30% predicted), respectively. Eight (16.3%) and six (12.2%) participants did not achieve their 80% (a presumed threshold for abnormal results) predicted normal values of their heart rate and O_2_-Pulse, respectively.

The pulmonary response to exercise was remarkable for a minute ventilation (V_E_) of 53.0 ± 16.5 L/min (56 ± 12% predicted) at peak exercise; only seven participants (14.3%) exceeded the 70% threshold for inappropriate ventilatory response to maximum effort exercise. The respiratory rate (RR) at peak exercise was below the 60 breaths/minute threshold (for possible concern for interstitial lung disease) in all participants. All participants achieved their anaerobic threshold (AT) as determined by V slope method on VO_2_. Additionally, ventilatory efficiency (ratio of V_E_ to carbon dioxide production [VCO_2_]) at peak exercise was 34.4 ± 9.8 (86 ± 20% predicted) with lowest V_E_/VCO_2_ at 31.5 ± 10.0 (79 ± 25% predicted). More comprehensive CPET data is available in Supplemental Table S2.

Thirty-five participants (71%) had RV/TLC > 0.35 and 43 participants (88%) had EFL at rest or during exercise (Table 2). Twenty-nine of the 35 participants with RV/TLC > 0.35 (83%) had EFL at rest or during exercise. Twenty-three of the 43 participants with EFL (53%) had EFL at rest and the remaining 20 (47%) developed EFL during exercise.

**Table 2:** Distributions of air trapping by the ratio of residual volume to total lung capacity (RV/TLC) and expiratory flow limitation (EFL)

	**RV/TLC > 0.35** ***N* = 35**	**RV/TLC ≤ 0.35** ***N* = 14**
**Expiratory flow limitation**		
**Had EFL at rest or during exercise**	29 (82.9%)	14 (100%)^a^
**Had EFL at rest**	19 (54.3%)	4 (28.6%)
**Developed EFL during exercise**	10 (28.6%)	10 (71.4%)
**Dynamic hyperinflation**		
**Had EELV increase across exercise**	14 (40.0%)	7 (50.0%)

*Footnote*: Distributions of air trapping as measured by residual volume to total lung capacity ratio (RV/TLC), expiratory flow limitation (EFL) as measured graphic overlap of tidal breathing with maximum expiratory flow-volume loop, and dynamic hyperinflation as measured by an increased in end-expiratory lung volume (EELV) from baseline throughout the exercise, estimated from regression coefficient of three EELV measurements at each of baseline (rest), 20%, 40%, 60%, and 80% of the load intensity (watts) of the peak exercise stage achieved. Values represent number of participants and their percent of the total number in each of the RV/TLC groups of > 0.35 or ≤ 0.35. See text for detailed methods

*Abbreviations*: *RV* residual volume, *TLC* total lung capacity, *EFL* expiratory flow limitation, *EELV* end-expiratory lung volume

^a^Having EFL at rest or during exercise was a required inclusion criteria for those participants with RV/TLC ≤ 0.35

### Efficacy of albuterol on exercise capacity, symptoms, quality of life, and physical activity

**Fig. 3:**
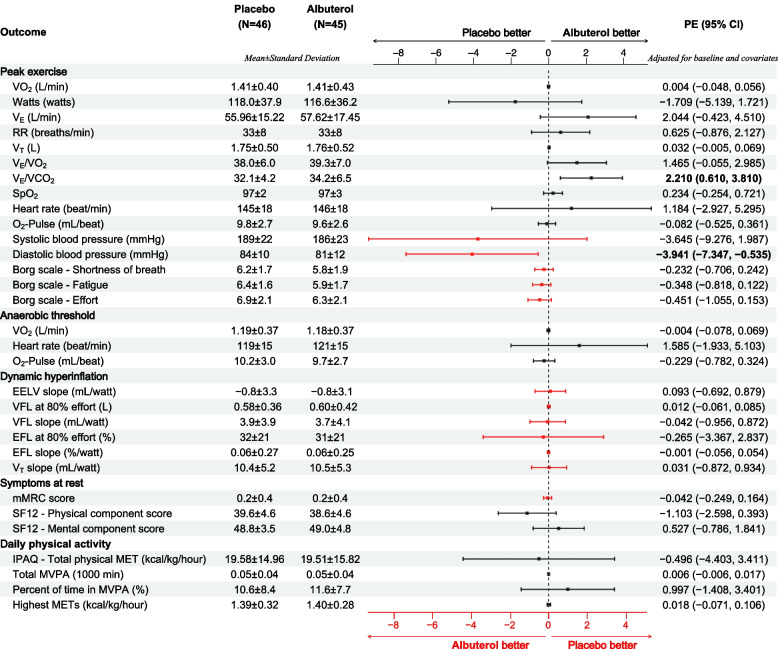
Associations of Albuterol and outcomes in all participants. The effect of Albuterol on the outcome variables was examined with a repeated measure design using mixed-effect linear regression modeling with the random subject effect and fixed effect variables including age, sex, height, weight, and the corresponding baseline measure of the outcome variable. The number of participants (N), the summary statistics (mean ± standard deviation) for each outcome variables measured in the placebo visit and the albuterol visit as well as the resulted parameter estimate (PE) representing the adjusted difference and the corresponding 95% confidence interval (CI) are shown. In this intention-to-treat analysis, N represents the number of participants who completed each (albuterol or placebo) arm of the study. The dot-and-whisker plots represent the PE and 95% CI with colors black (scaled on the top) and red (scaled on the bottom) to distinguish outcomes in which higher versus lower values are preferable. The PE and 95% CI for the statistically significant associations were shown in bold. Abbreviations: VO_2_: oxygen uptake; Watts: work stage completed in watts; V_E_: minute ventilation value; RR: respiratory rate; V_T_: tidal volume; VCO_2_: carbon dioxide production; SpO_2_: oxygen saturation; O_2_-Pulse: oxygen uptake per heartbeat; EELV: end-expiratory lung volume; VFL: volume of the tidal breath that is flow limited on expiration; EFL: expiratory flow limitation; SF12: Short Form 12-Item Health Survey; IPAQ: International Physical Activity Questionnaire; MET: metabolic equivalent: MVPA: moderate to vigorous physical activities; PE: parameter estimate; CI: confidence interval

In the primary analysis with inclusion of data from all 49 participants, there was no significant change in primary or any of the secondary outcomes by intention-to-treat or per-protocol analysis (Fig. 3). Although V_E_/VCO_2_ and diastolic blood pressure (DBP) at peak exercise showed a significant improvement with albuterol, these variables were not a priori included as a secondary outcome. Analysis including only those participants who completed the entire study (*n* = 42) showed similar result with no significant changes in primary or a priori secondary outcomes (Supplemental Fig. S1).

**Fig. 4:**
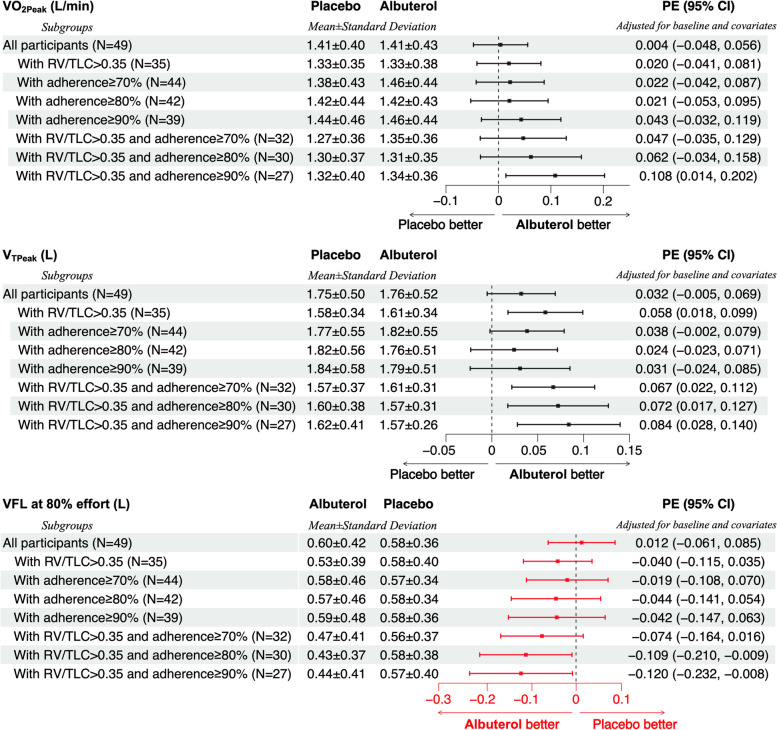
Associations of Albuterol and outcomes in subgroup analysis. The effect of Albuterol on the outcome variables was examined with a repeated measure design using mixed-effect linear regression modeling with the random subject effect and fixed effect variables including age, sex, height, weight, and the corresponding baseline measure of the outcome variable. The analysis was separately done within the whole group of participants and the subgroups based on RV/TLC and/or adherence. The number of participants (N), the summary statistics (mean ± standard deviation) for each outcome variables measured in the placebo visit and the albuterol visit as well as the resulted parameter estimate (PE) representing the adjusted difference and the corresponding 95% confidence interval (CI) are shown. In these subgroup analyses, N represents the number of participants in both arms of the study who met the air trapping and/or adherence criteria. The dot-and-whisker plots represent the PE and 95% CI with colors black (scaled on the top) and red (scaled on the bottom) to distinguish outcomes in which higher versus lower values are preferable. Abbreviations: VO_2Peak_: oxygen uptake in peak exercise; V_TPeak_: tidal volume in peak exercise; VFL: volume of the tidal breath that is flow limited on expiration; PE: parameter estimate; CI: confidence interval

Subgroup analysis based on presence of RV/TLC > 0.35 and/or level of adherence to treatment protocol showed a gradually larger improvement in primary outcome (peak VO_2_ response) that became statistically significant with increasing adherence to assigned treatment (Fig. 4). Similar gradual improvement in some of the secondary outcomes was also observed (Fig. 4).

**Fig. 5:**
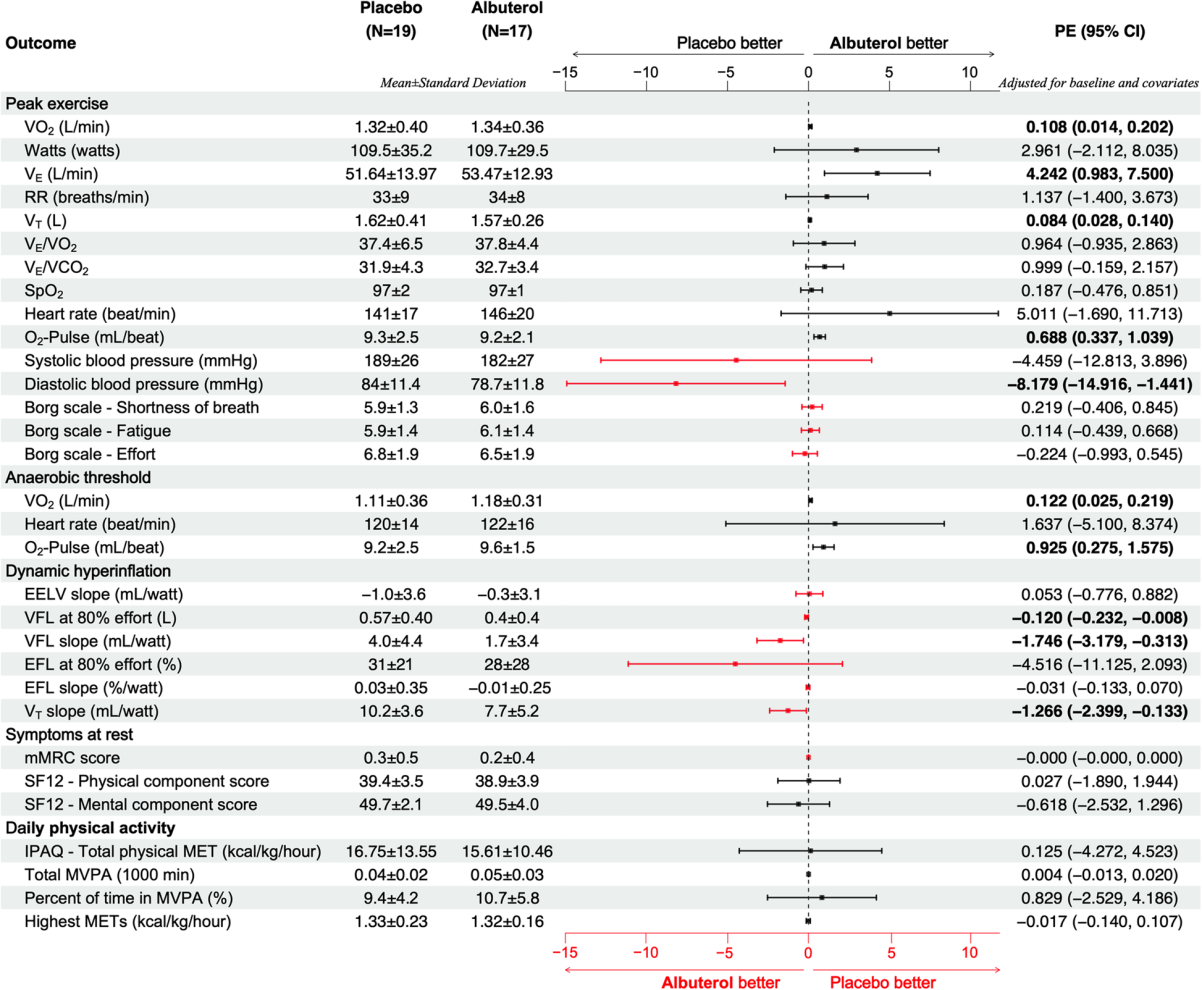
Associations of Albuterol and outcomes in the subgroup with RV/TLC > 0.35 and ≥ 90% adherence. The effect of Albuterol on the outcome variables was examined with a repeated measure design using mixed-effect linear regression modeling with the random subject effect and fixed effect variables including age, sex, height, weight, and the corresponding baseline measure of the outcome variable. The number of participants (N), the summary statistics (mean ± standard deviation) for each outcome variables measured in the placebo visit and the albuterol visit as well as the resulted parameter estimate (PE) representing the adjusted difference and the corresponding 95% confidence interval (CI) are shown. In this subgroup analysis, N represents the number of participants in each arm of the study who met the adherence criteria of ≥ 90%. The dot-and-whisker plots represent the PE and 95% CI with colors black (scaled on the top) and red (scaled on the bottom) to distinguish outcomes in which higher versus lower values are preferable. The PE and 95% CI for the statistically significant associations were shown in bold. Abbreviations: VO_2_: oxygen uptake; Watts: work stage completed in watts; V_E_: minute ventilation value; RR: respiratory rate; V_T_: tidal volume; VCO_2_: carbon dioxide production; SpO_2_: oxygen saturation; O_2_-Pulse: oxygen uptake per heartbeat; EELV: end-expiratory lung volume; VFL: volume of the tidal breath that is flow limited on expiration; EFL: expiratory flow limitation; SF12: Short Form 12-Item Health Survey; IPAQ: International Physical Activity Questionnaire; MET: metabolic equivalent: MVPA: moderate to vigorous physical activities; PE: parameter estimate; CI: confidence interval

In per-protocol subgroup analysis of participants with RV/TLC > 0.35 and ≥ 90% adherence (*n* = 27), albuterol inhalation caused a significant improvement in primary outcome (peak VO_2_) (parameter estimate [95% confidence interval] = 0.108 [0.014, 0.202]) as well as some of the secondary outcomes: tidal volume (V_T_) (0.084 [0.028, 0.140]), minute ventilation (V_E_) (4.242 [0.983, 7.500]), oxygen-pulse (O_2_-Pulse) (0.688 [0.337, 1.039]), and some of the dynamic hyperinflation indices (Fig. 5). However, no significant changes in physical activity, symptoms, or quality of life scores were observed (Fig. 5).

Similar findings were observed for primary and subgroup analysis when paired t-test approach was used to examine the response among those participants who completed both arms of the study (Supplemental Table S3). Inclusion of other lung function indices representing small airway disease (FEF_25-75_ or FEF_75_) or air trapping did not show any significant interaction with the effect of albuterol on the study outcomes.

## Discussion

In this clinical trial, we found that inhaled bronchodilator treatment with albuterol did not improve exercise capacity (VO_2_, primary outcome) or other exercise performance indices, respiratory symptoms, quality of life, or daily physical activity (secondary outcomes) in nonsmoking SHS tobacco-exposed individuals with preserved spirometry but with air trapping (defined by RV/TLC > 0.35 or presence of EFL). The lack of efficacy was present regardless of how well the participants adhered to the inhalational treatment protocol (per-protocol analysis by ≥ 70%, ≥ 80%, or ≥ 90% adherence). However, in subgroup analysis of those with air trapping defined by an RV/TLC > 0.35 who had ≥ 90% adherence to treatment, inhaled albuterol did cause an improvement in exercise capacity (VO_2_, primary outcome), some measures of dynamic hyperinflation, ventilation, and oxygen-pulse (a proxy of cardiac output); however, symptoms, quality of life, or daily physical activity level were not improved. Of note, inhaled albuterol also improved the diastolic blood pressure at peak exercise, an outcome that was not specified a priori in our study, but retrospectively was of interest given the consideration that air trapping may adversely contribute to cardiovascular diastolic dysfunction [9, 43, 44].

Occupational exposure to SHS, a risk factor for development of COPD [1–3], is associated with both reduced diffusing capacity [4, 45] and wide distributions of lung volume indices, such as RV/TLC or FRC/TLC, that represent air trapping despite preserved spirmtery [4, 5]. Studies have shown that even in the absence of spirometric airflow obstruction, elevated RV/TLC or FRC/TLC can identify a subset of individuals with worse exercise capacity and symptoms who may progress to develop overt airflow obstruction and spirometric COPD [5–8]. Thus, air trapping in the setting of preserved spirometry may represent an early small airway disease, which contributes to exercise limitation and symptoms, probably through dynamic hyperinflation, even in the absence of spirometric airflow obstruction [5, 45]. In such a case, bronchodilator treatment may be useful in relieving air trapping and dynamic hyperinflation and thus improving exercise capacity.

Although our findings in this study are not supportive of such hypothesis, in the subgroup with RV/TLC > 0.35 and high adherence to treatment, bronchodilator use with albuterol did improve exercise capacity and dynamic hyperinflation, suggesting that at least in those with significant air trapping, as measured by elevated RV/TLC, bronchodilator could be advantageous. However, further research, perhaps in people with preserved spirometry but more significant air trapping, is needed to confirm this possibility. Compared to people with history of occupational SHS exposure and no direct smoking, people with history of direct smoking have a much wider distribution of lung volumes representing air trapping with approximately a third showing abnormal RV/TLC greater than the upper limit of predicted normal values [5–7]. Thus, it is plausible that inhaled bronchodilator treatment could have a larger effect in those with history of direct smoking and preserved spirometry who have significant air trapping. A recent a randomized controlled trial on the utility of bronchodilators in direct tobacco-exposed persons with preserved spirometry who had respiratory symptoms found that bronchodilator treatment did not alleviate respiratory symptoms [46]. However, lung volumes were not assessed in that study, and thus stratification of the participants by their lung volumes or air trapping could not be done, making it impossible to distinguish smokers with preserved spirometry with abnormal/high RV/TLC or FRC/TLC who may have been more likely to benefit from bronchodilators.

During the course of this study, we recruited and enrolled nonsmoking participants with preserved spirometry and air trapping. To improve recruitment, we enrolled participants with air trapping defined as (1) those with RV/TLC > 0.35, a limit that we had previously determined as a reasonable cutoff for presence of exercise limitation and symptoms [5], but also (2) those with EFL as determined by presence of graphic overlap of tidal breathing on maximum expiratory flow-volume loop at rest or during exercise. EFL has been shown to be associated with air trapping and dynamic hyperinflation, both of which contribute to exercise limitation and worse respiratory symptoms [5, 47–49]. Two interesting observations from our study in this regard are that in this non-obese population at risk for COPD due to occupational SHS exposure but with preserved spirometry, about 20% of those with RV/TLC > 0.35 did not have any EFL at rest or during exercise, while some with RV/TLC ≤ 0.35 had EFL even at rest. Given the subgroup analysis finding of albuterol effectiveness in those with RV/TLC > 0.35 and high adherence to therapy, these observations suggest resting lung volumes that represent air trapping such as RV/TLC may be better predictors of performance response to bronchodilator treatment than the presence of EFL at rest or during exercise. These findings are consistent with our previous studies in implicating lung volumes as better prognostic indicators for lung disease presence in pre-COPD when flows are within normal limits.

One interesting finding in our study was the decline in diastolic blood pressure that was observed in response to albuterol administration. While change in diastolic blood pressure was not a priori hypothesized as an outcome of albuterol therapy, there are potential mechanistic rationales that could explain the observed effect. In patients with diastolic heart failure (heart failure with preserved ejection fraction or HFpEF), both intravenous administration of dobutamine (a β-agonist) and inhalational administration of albuterol (2.5 mg via nebulization, which is roughly equivalent to four 90 mcg inhalation via metered dose inhaler) have been shown to elicit pulmonary and systemic vasodilatory effects along with a decline in systolic and diastolic blood pressure [50–52]. These studies however did not include any characterization of pulmonary status of the participating subjects. On the other hand, HFpEF has been described in patients with COPD whether severe [53] or mild [54] and even in those with preserved spirometry (normal FEV_1_/FVC) with declining vital capacity, as it happens with higher RV and RV/TLC and air trapping [55]. Given the above, another potential explanation for these observations could be the presence of air trapping. Lungs occupy the same finite space (thoracic cavity) as heart and great vessels, and increased lung volumes as it happens with air trapping and dynamic hyperinflation could introduce space limitation and changes to intrathoracic pressure, which in turn could contribute to cardiovascular dysfunction [9, 56]. Studies have described arterial stiffness as well as abnormalities in myocardial wall motion and relaxation in presence of abnormal lung volumes and air trapping [55, 57], all of which could contribute to increased ventricular end-diastolic pressure and hence increased diastolic blood pressure. Therefore, the changes in diastolic blood pressure seen with albuterol treatment in our study may have their origin in the effects of albuterol on lung volumes in addition to any direct effects on vasculature.

Our study has limitations that should be kept in view. First, the study sample size was small and further suffered from the research holds applied during the COVID-19 pandemic. Given that the final number of participants was significantly fewer than what was proposed in the original sample size and power calculation, the study was underpowered to detect the changes in the primary outcome. However, while the main study findings were negative, the subgroup analyses did provide interesting evidence suggesting the usefulness of bronchodilator therapy in the context of preserved spirometry with air trapping in individuals at risk for COPD. The findings also suggest that the nature of lung volume indices representing air trapping in the setting of preserved spirometry may in fact be an airway process not captured by airflow measures but observed on lung volume measurements. Second, studying the nature of air trapping in the setting of preserved spirometry may have been better performed in a population with more severe air trapping but preserved spirometry. Such population may be studied by enrolling individuals with history of direct smoking who have preserved spirometry but significant air trapping. However, the purpose of the current study was to examine the nonsmoking population with history of occupational SHS exposure. Epidemiologic data suggest that nearly 21% of the US population continue to be exposed to tobacco smoke [58], and thus understanding the health effects associated with exposure to SHS, which is a COPD risk factor, is of significant public health importance. Understanding whether inhaled bronchodilators could have beneficial effects on performance of respiratory system and exercise capacity could provide rationale for such therapy for when the respiratory demands are similarly increased during diseases such as respiratory infections. Third, we used albuterol, a short-acting β-agonist bronchodilator (SABA), instead of a long-acting or an ultra-long-acting β-agonist bronchodilator (LABA or ULABA) to test our hypothesis. Routine use of albuterol at high doses (800 mcg per day on four times a day dosing) in people with asthma has been reported to be associated with increased airway hyperresponsiveness as well as tachyphylaxis [59–62], although other studies have also documented opposing results showing that the airway bronchodilator responses are maintained even with high doses of albuterol [63–65]. However, the overall evidence in the literature suggests that in those without history of asthma or COPD, the lower doses and frequencies utilized in our study should not dampen the bronchodilator response. Moreover, such dampening of response would have resulted in a bias towards null hypothesis. Furthermore, while using LABA or ULABA may have been a more superior choice for the purpose of this study, we settled on using a short-acting agent in this study population with no overt obstruction because of concerns about adherence to therapy as those with milder symptoms or lung function abnormalities might be more likely to discontinue the use of study medication with occurrence of any mild adverse effects.

## Conclusions

In conclusion, we did not find a significant improvement from albuterol on exercise performance, respiratory symptoms, quality of life, or daily physical activity level in this population at risk for COPD due to occupational exposure to SHS who had preserved spirometry but also air trapping and/or expiratory airflow limitation. However, in the subset of those participants in whom air trapping was strictly defined by RV/TLC > 0.35, albuterol did improve exercise performance and some measures of dynamic hyperinflation although no other outcomes. This study suggests that air trapping in the setting of pre-COPD (tobacco-exposed persons with preserved spirometry) may be related to small airway disease that is not considered significant by spirometric indices of airflow obstruction. In addition, it suggests that stratification of the tobacco-exposed persons with preserved spirometry by lung volumes and air trapping may help in identifying a subset who do benefit from the use of bronchodilators. Further study of this approach in persons with history of direct smoking and preserved spirometry who may have more severe air trapping should be able to provide a clinically useful indication in that population.

### Supplementary Information

**Additional file 1.**

## Data Availability

The datasets generated and/or analyzed during the current study are available from the corresponding author on reasonable request.

## Abbreviations

AQ20: Airway Questionnaire 20
BP: Blood pressure
CAT: COPD Assessment Test
COPD: Chronic obstructive pulmonary disease
CPET: Cardiopulmonary exercise testing
DBP: Diastolic blood pressure
EELV: End-expiratory lung volume
EFL: Expiratory flow limitation
FAMRI: Flight Attendant Medical Research Institute
FEF_25-75_: Forced expiratory flow at 25% to 75% of FVC
FEF_75_: Forced expiratory flow at 75% of FVC
FEV_1_: Forced expiratory volume in 1 s
FRC: Functional residual capacity
FVC: Forced vital capacity
GLI: Global Lung Function Initiative
GOLD: Global Initiative on Obstructive Lung Disease
HR: Heart rate
IC: Inspiratory capacity
IPAQ: International Physical Activity Questionnaire
LLN: Lower limit of normal
MEF: Maximum expiratory flow
mMRC: Modified Medical Research Council Dyspnea Scale
O_2_-Pulse: Oxygen-pulse
PFT: Pulmonary function test
RR: Respiratory rate
RV: Residual volume
SF-12: Short Form 12-Item Health Survey
SHS: Secondhand tobacco smoke
SpO_2_: Oxygen saturation
TLC: Total lung capacity
ULN: Upper limit of normal
VA: Alveolar volume
VCO_2_: Carbon dioxide production
V_E_: Minute ventilation
V_FL_: Volume of the tidal breath that is flow-limited on expiration
VO_2_: Oxygen consumption
V_T_: Tidal volume
Watts_Peak_: Peak work achieved in watts
Work_Total_: Cumulative work achieved in Watts-Minutes throughout the exercise
