# Supplementary material for: Effect of twice daily inhaled albuterol on cardiopulmonary exercise outcomes, dynamic hyperinflation, and symptoms in secondhand tobacco-exposed persons with preserved spirometry and air trapping: a randomized controlled trial
Source: BMC Pulm Med. 2024 Jan 20;24:44. doi: 10.1186/s12890-023-02808-7 (PMC10799390; doi:10.1186/s12890-023-02808-7)
Supplement: Supplementary file 1 — Additional file 1. [file 12890_2023_2808_MOESM1_ESM.pdf]

SUPPLEMENTARY APPENDIX

Effect of twice daily inhaled albuterol on cardiopulmonary exercise outcomes, dynamic hyperinflation, and symptoms in secondhand tobacco-exposed persons with preserved spirometry and air trapping: a randomized controlled trial

Running title: Bronchodilators for treatment of air trapping sequalae in pre-COPD

Siyang Zeng, MS [1,2,3], Melissa Nishihama, BS [2,3], Lemlem Weldemichael, MD [2,3], Helen Lozier, BS [2,3,4], Warren M Gold, MD [3], Mehrdad Arjomandi MD [2,3,5]

Affiliations:
[1] Department of Biomedical Informatics and Medical Education, University of Washington, Seattle, WA, USA
[2] Pulmonary and Critical Care Section, San Francisco Veterans Affairs Medical Center, San Francisco, California, USA
[3] Division of Pulmonary and Critical Care Medicine, Department of Medicine, University of California, San Francisco, California, USA
[4] Carver College of Medicine, University of Iowa, Iowa City, Iowa, USA
[5] Division of Occupational and Environmental Medicine, Department of Medicine, University of California, San Francisco, California, USA

TABLE OF CONTENT:

DETAILED METHODS **2**
SECONDHAND TOBACCO SMOKE EXPOSURE CHARACTERIZATION 2
PULMONARY FUNCTION TESTING (PFT) 2
CARDIOPULMONARY EXERCISE TESTING (CPET) 3
EXPIRATORY FLOW LIMITATION AND END EXPIRATORY LUNG VOLUME MEASUREMENTS DURING EXERCISE 4
SUPPLEMENTAL TABLES **7**
SUPPLEMENTAL FIGURE **18**
REFERENCES **20**

DETAILED METHODS

Secondhand tobacco smoke Exposure Characterization

Secondhand tobacco smoke (SHS) exposure was characterized by an exposure questionnaire that was developed by our UCSF Flight Attendant Medical Research Institute (FAMRI) Center of Excellence,[1] and modified to acquire information on airline-related occupational history, as described previously.[2,3] Briefly, this included employer airlines, duration of employment, and flight routes (domestic vs. international) with quantification of "cabin SHS exposure" as the number of years of employment before the smoking ban, the period during which the flight crew were exposed to SHS in the aircraft cabin. Other possible sources of exposure to SHS (non-cabin SHS exposure) were also explored by questioning participants about their non-cabin SHS exposure in additional settings, as described previously.[4]

Pulmonary Function Testing (PFT)

Routine PFTs were performed in the seated position using a model Vmax 229 CareFusion (CareFusion Corp., Yorba Linda, CA) and nSpire body plethysmograph (nSpire Health Inc., Longmont, CO). This included measurement of the low-volume curve and spirometry,[5] lung volume by single breath dilution,[6,7] and plethysmography,[8] airway resistance during panting at functional residual capacity (FRC),[9,10] and single breath carbon monoxide diffusing capacity.[11] Air trapping, which is inferred from an increased in residual volume (RV), was quantified using RV to total lung capacity (TLC) ratio (RV/TLC). Pulmonary function studies were conducted according to the American Thoracic Society (ATS) and European Respiratory Society (ERS) guidelines.[12-17] Participants did not undergo bronchodilator administration.

Cardiopulmonary Exercise Testing (CPET)

Participants performed physician-supervised, symptom-limited, progressively increasing stepwise maximal exercise tests in the seated position on an electromagnetically braked, seated cycle ergometer (Corvial cpet, Lode B.V., Groningen, The Netherlands). Participants were encouraged to give their best effort, and during testing, were encouraged to continue exercise until a VO_2 plateau effect on a breath-to-breath analysis of oxygen consumption was visually observed; however, they were advised that they could stop voluntarily at any time they believed they could not continue. We continuously monitored heart rate (HR), blood pressure (BP), electrocardiogram (ECG), and breath-by-breath gas exchange.

The protocol consisted of 3-min rest, 1-min unloaded (freewheeling) cycling at 60 rpm, followed by increasing work rate of 20 to 40 Watts per step at 2-minute intervals to the maximum tolerated, and 5-min of recovery. Twelve lead ECGs were monitored continuously; ECGs and blood pressure (measured manually by a physician with a cuff) were recorded every 2 min. Oxyhemoglobin saturation (SpO_2 or O_2sat), determined by pulse oximetry, was recorded continuously.

Minute ventilation (V_E), oxygen uptake (VO_2) and carbon dioxide output (VCO_2) were measured breath-by-breath with an open-circuit metabolic cart (model Vmax 229, CareFusion, Yorba Linda, CA). The volumes of the flow meter, mouthpiece, and filter (70 mL x breathing frequency) were subtracted from V_E for the V_E/VCO_2 calculations. Anaerobic threshold (AT) was determined by the V-slope method.[18,19] Immediately before all tests, the gas analyzers were calibrated using reference gases of known concentrations and the ventilometer was calibrated using a 3-liter syringe (Hans Rudolph, Kansas, MO). The metabolic system was verified using

four trained technicians who provided monthly exercise values as biological standards for the Laboratory.

Expiratory Flow Limitation and End Expiratory Lung Volume Measurements during Exercise

To determine whether dynamic hyperinflation contributed to the level of maximum exercise achieved, the participants underwent a separate dynamic hyperinflation exercise testing protocol for measurement of end-expiratory lung volume (EELV) and expiratory flow limitation (EFL) approximately 45 minutes after completing their maximum effort exercise testing. This protocol included tidal volume (V_T), inspiratory capacity (IC), and maximal expiratory flow (MEF) measurements in seated position at 2-minute steps with increasing work rates corresponding to 20%, 40%, 60%, and 80% of the peak VO_2 they attained during the maximum effort exercise testing, as described by O'Donnell et al.[20,21] Measurements of IC and MEF were made three times during the second minute at each step and work rate while recording tidal flow-volume loops to assess for evidence of expiratory flow limitation. The volume of the tidal breath that is flow-limited on expiration (V_{FL}) was measured on flow-volume loop graphics using ImageJ (version 1.44, NIH, Bethesda, MD, USA) and used to calculate %EFL according to formula $[(V_{FL}/V_T)*100]$ at each work rate. EELV and RV (TLC- IC) were similarly calculated using ImageJ at each work rate.

Physical Activity Monitoring using Actigraphy

Physical activity was monitored using a triaxial accelerometer-based activity monitor (ActiGraph GT3X, Actigraph Corporations, Pensacola, FL) as described previously.[22] Accelerometer data was recorded in 10-second epochs and in the three "vertical", "horizontal",

and "perpendicular" axes representing forward, sideways, and upward/downward motion, respectively.

The ActiGraph monitor was initialized to continuously collect data over a seven-day period. It was then mailed to participants along with a daily diary to keep a log of the time the monitor was worn and the activities the participants performed during that time. All participants received the ActiGraph monitors at least seven days prior to the in-laboratory visit, during which data on respiratory symptom questionnaire, PFT and CPET was collected. Participants were instructed to wear the ActiGraph monitor on the waist upon awakening using a waist band provided and to keep it on continuously for at least eight hours for seven consecutive days beginning the first day of their work week and beginning the start of their day. The seven-day monitoring was chosen to allow for adequate number of weekday data collection.[23] All participants were carefully instructed on how the device should be positioned.

Actigraphy data was processed using the ActiLife software program (Version 6.11.9; ActiGraph LLC) and saved in raw format as GT3X files. The ActiLife software generates a total of 52 variables in the distance, time (activity and sedentary) and energy domains. ActiLife outputs use direct measurement of distance, steps, and time for quantifying activity, but quantifying of energy expenditure (EE) requires translation of activity using different prediction models.

Actigraphy data had a repeated measure design as each participant wore the monitor for seven consecutive days in the week prior to their second and third visits. Thus, the data was further summarized into seven-day average values (weekly "mean" values) and the highest values of "maximum" values (maximum per epoch) across all seven days (weekly "highest" values). The actigraphy data was matched against the diary to ascertain the appropriate usage of

the monitor, and the data was considered to be acceptable if the participants wore the monitor for a minimum of 3 days and for greater than 5 hours (300 minutes) per day. The total amount of the time that the monitor was worn was included in the regression models as *total time worn*.

A final set of variables selected based on a combination of the machine learning and literature-guided approaches to provide meaningful variables of highest predictive value for our proposed analysis was used for the purpose of this analysis as described previously.[22]

Respiratory Symptom Assessment

Patient-reported respiratory symptoms, physical activity, and quality of life assessments were conducted using the COPD Assessment Test (CAT),[24] modified Medical Research Council (mMRC) Dyspnea Scale,[25] the Short Form 12-Item Health Survey (SF-12),[26] International Physical Activity Questionnaire (IPAQ),[27] Airway Questionnaire 20 (AQ20),[28] and a self-reported questionnaire (UCSF Flight Attendant Medical Research Institute FAMRI SHS Questionnaire) that elicited symptoms of dyspnea, cough, and participants' perception of a decreased level of exertion compared to peers over the year preceding enrollment.[1] A dichotomous indicator of respiratory symptoms was defined by mMRC ≥1 or report of at least one respiratory symptom on the UCSF FAMRI SHS Questionnaire. A dichotomous cause of exercise cessation (dyspnea versus fatigue or effort; $Dyspnea_{Peak}$) was determined based on the highest score reported by the participants at the end of the maximum effort exercise testing using the modified Borg Rating of Perceived Exertion (Borg), with the Category-Ratio Scale anchored at number 10 (CR10).[29]

SUPPLEMENTAL TABLES

Table S1- Participant characteristics at baseline for the groups.

Participant characteristics	Participants completed the study (N=42)	Participants with available data (N=49)	Participants with air trapping and 90% adherence (N=27)
**Demographics and anthropometrics**			
Age (years)	66.0±7.8	66.2±8.0	67.1±8.2
Female sex [n (%)]	38 (90.5%)	43 (87.8%)	26 (96.3%)
Height (cm)	166.4±7.7	166.4±8.1	164.9±7.3
Weight (kg)	65.9±11.8	66.9±12.9	63.0±11.6
BMI (kg/m^2)	23.7±3.2	24.0±3.7	23.1±3.4
Hemoglobin (g/dL)	13.5±0.4	13.5±0.4	13.4±0.2
Having EFL	15 (35.7%)	17 (34.7%)	11 (40.7%)
**SHS Exposure**			
Ever Cabin SHS exposure [n (%)]	34 (81.0%)	41 (83.7%)	21 (77.8%)
Cabin SHS exposure among exposed (years)	18.0±8.9	17.6±9.0	19.4±8.4
Any form of non-cabin SHS exposure [n (%)]	42 (100%)	49 (100%)	27 (100%)
Childhood home SHS exposure [n (%)]	25 (59.5%)	29 (59.2%)	17 (63.0%)
Adult home SHS exposure [n (%)]	19 (45.2%)	21 (42.9%)	12 (44.4%)
Non-airline occupational SHS exposure [n (%)]	35 (83.3%)	42 (85.7%)	24 (88.9%)
Other SHS Exposure [n (%)]	37 (88.1%)	43 (87.8%)	24 (88.9%)
**Symptoms**			
mMRC Dyspnea Scale ≥1 [n (%)]	6 (27.3%)	10 (34.5%)	5 (31.3%)
SF12			
Physical component score	38.2±4.9	38.5±4.9	39.4±3.3
Mental component score	48.6±4.7	48.9±4.6	49.1±4.0
IPAQ score			
High [n (%)]	37 (88.1%)	43 (87.8%)	23 (85.2%)
Moderate [n (%)]	5 (11.9%)	5 (10.2%)	4 (14.8%)
Low [n (%)]	0 (0%)	1 (2.0%)	0 (0%)
CAT	6.22±5.34	6.60±5.68	5.67±4.66
Participants with any respiratory symptoms [n (%)]	40 (95.2%)	45 (91.8%)	25 (92.6%)
Participants ever experiencing shortness of breath [n (%)]	11 (26.2%)	13 (26.5%)	7 (25.9%)

Participants with cough ≥2 episodes/year [n (%)]	40 (95.2%)	44 (89.8%)	25 (92.6%)
Participants with less activity than peers [n (%)]	3 (7.1%)	4 (8.2%)	2 (7.4%)
**Pulmonary Function Tests**			
FEV_1 (% predicted)	103±16	103±16	99±12
FVC (% predicted)	107±16	107±16	103±11
FEV_1/FVC	0.75±0.05	0.75±0.05	0.75±0.05
FEV_1/FVC (% predicted)	96±6	96±6	95±8
FEF_{25-75} (% predicted)	98±32	98±30	95±35
FEF_{75} (% predicted)	124±56	127±56	132±69
D_{LCO} adjusted for Hgb (mL/min/mmHg)	21.6±4.2	21.4±4.3	22.0±4.53
D_{LCO} adjusted for Hgb (% predicted)	84±11	84±12	87 ±11
D_L/V_A adjusted for Hgb (mL/min/mmHg/L)	4.45±0.58	4.36±0.61	4.56±0.62
D_L/V_A adjusted for Hgb (% predicted)	89±11	88±12	92±11
Alveolar volume (V_A) (L)	4.90±0.86	4.97±1.00	4.71±0.66
Alveolar volume (V_A) (L) (% predicted)	92±12	93±12	91±9
TLC (% predicted)	101±11	101±11	101±9
RV (% predicted)	99±15	98±16	105±12
RV/TLC (%)	39±7	39±7	42±5
RV/TLC (% predicted)	95±14	94±13	101±10
FRC (% predicted)	96±16	96±17	99±16
FRC/TLC (%)	51±7	51±7	53±7
FRC/TLC (% predicted)	92±12	92±13	95 ±13
**Cardiopulmonary Testing Measurements**			
VO_{2Peak} (L/min)	1.32±0.38	1.33±0.41	1.27±0.38
VO_{2Peak} (% predicted)	101±22	99±21	104±22
$VO_{2Peak/kg}$ (mL/min.kg)	20.2±4.9	20.0±4.8	20.3±5.1
$VO_{2Peak/kg}$ (% predicted)	86±20	86±20	89±22
RER_{Peak}	1.13±0.09	1.13±0.08	1.12±0.07
RER_{Peak} (% predicted)	103±8	103±8	102±6
VCO_{2Peak} (L/min)	1.50±0.46	1.52±0.50	1.43±0.45
$Watts_{Peak}$ (watts)	112±32	113±36	106±28
$Watts_{Peak}$ (% predicted)	114±27	115±28	119±34
Cumulative work (watt-minute)	516±233	535±263	501±212
Stages completed	4.8±0.9	4.9±1.0	5.0±0.9
Total duration (minute)	9.6±1.9	9.8±2.0	9.9±1.8
Symptoms at peak exercise (Borg Scale 0 to 10)			
Shortness of Breath	5.49±1.87	5.60±1.85	5.52±1.67
Effort	6.17±2.06	6.29±2.06	6.00±1.80
Fatigue	5.66±1.80	5.83±1.88	5.67±1.71
**Pulmonary Response**			
V_{EPeak} (L/min)	52.7±15.5	53.0±16.5	49.1±11.8
V_{EPeak} (% predicted)	57±13	56±12	56±13

RR_{Peak} (breaths/min)	31.2±6.7	30.6±6.7	31.2±7.2
V_{TPeak} (L)	1.71±0.45	1.77±0.57	1.61±0.34
V_{TPeak} (% predicted)	94±18	96±19	94±18
V_E/VO_{2Peak}	39.1±12.1	38.8±11.3	38.3±12.7
V_E/VCO_{2Peak}	34.9±10.4	34.4±9.8	34.2±11.7
V_E/VCO_{2Peak} (% predicted)	87±26	86±24	86±29
VO_2 at Anaerobic Threshold (VO_{2AT}) (L/min)	1.11±0.33	1.11±0.34	1.11±0.32
VO_2 at Anaerobic Threshold (VO_{2AT}) (% predicted)	81±22	81±23	86±23
VO_2 at Anaerobic Threshold (VO_{2AT}) (% of VO_{2Peak} achieved)	86±19	86±20	88±18
V_E/VCO_2 at Anaerobic Threshold	35.3±14.7	35.2±13.7	36.3±17.5
**Cardiovascular Response**			
HR_{Rest} (beat/min)	75±15	76±19	80±23
HR_{Peak} (beat/min)	141±23	141±22	142±20
HR_{Peak} (% predicted)	92±14	92±14	93±13
HR_{Peak}≤90% predicted [n (%)]	17 (40.5%)	21 (42.9%)	11 (40.7%)
HRR	12.7±21.4	12.8±21.0	11.3±18.8
HRR≥15 [n (%)]	19 (45.2%)	23 (46.9%)	11 (40.7%)
HR_{Slope} (beat/min/10 Watts)	6.44±2.05	6.30±2.06	6.22±1.71
SBP_{Rest} (mmHg)	128±18	128±17	129±19
SBP_{Peak} (mmHg)	186±28	189±29	187±31
SBP_{Slope} (mmHg/10 Watts)	5.60±1.87	5.87±2.13	5.71±2.12
DBP_{Rest} (mmHg)	73±10	73±9	72±11
DBP_{Peak} (mmHg)	83±11	84±11	83±11
DBP_{Slope} (mmHg/10 Watts)	0.98±1.08	1.07±1.11	1.10±1.24
HRE (met at least one criterion) [n (%)]	22 (75.9%)	12 (60.0%)	34 (69.4%)
Rise in SBP ≥50 mmHg (≥60 mmHg for men)	20 (69.0%)	11 (55.0%)	31 (63.3%)
SBP_{Peak} ≥190 mmHg (≥210 mmHg for men)	15 (51.7%)	8 (40.0%)	23 (46.9%)
DBP_{Peak} ≥105 mmHg	0 (0%)	0 (0%)	0 (0%)
O_2-$Pulse_{Peak}$ (mL/beat)	9.64±3.62	9.74±3.80	9.11±2.76
O_2-$Pulse_{Peak}$ (% predicted)	107±32	106±30	108±30
O_2-$Pulse_{Rest}$ (mL/beat)	3.49±0.94	3.54±1.18	3.45±0.98
O_2-$Pulse_{Slope}$ (mL/beat/10 Watt)	0.50±0.20	0.50±0.19	0.49±0.18
O_2-Pulse at Anaerobic Threshold (mL/beat)	9.07±2.67	9.15±3.04	9.08±2.57
O_2-Pulse at Anaerobic Threshold (% O_2-$Pulse_{Peak}$)	97±20	98±21	101±19
SpO_{2Rest}	97.7±1.9	97.5±1.8	97.5±2.0
SpO_{2Peak}	97.4±1.6	97.3±1.5	97.3±1.5
Change in SpO_2 at peak exercise	-0.29±1.44	-0.20±1.35	-0.26±1.68
**Dynamic Hyperinflation**			

VFL_{Rest} (L)	0.19±0.25	0.20±0.25	0.23±0.26
$VFL_{80\%\ effort}$ (L)	0.54±0.35	0.55±0.36	0.54±0.36
VFL_{Slope} (mL/watt)	3.71±3.50	3.80±3.38	3.41±3.23
EFL_{Rest} (%)	19.4±25.6	19.5±24.7	21.6±24.7
$EFL_{80\%\ effort}$ (%)	29.8±18.9	29.7±18.4	31.2±20.6
EFL_{Slope} (%/watt)	0.091±0.247	0.091±0.230	0.084±0.223
EFL at rest [n (%)]	19 (45.2%)	23 (46.9%)	14 (51.9%)
EFL at rest or during exercise [n (%)]	36 (85.7%)	43 (87.8%)	22 (81.5%)
$EELV_{Rest}$	2.83±0.55	2.82±0.64	2.88±0.56
$EELV_{80\%\ effort}$	2.78±0.52	2.76±0.59	2.80±0.47
Slope of EELV across exercise stages	-0.59±3.89	-0.69±3.74	-1.09±2.66
No. with increase in EELV slope [n (%)]	19 (45.2%)	21 (42.9%)	11 (40.7%)

Footnote: Demographics, secondhand smoke (SHS) exposure, symptoms, and lung function in participants with preserved spirometry that underwent exercise testing. Other SHS exposure was defined as non-aircraft cabin SHS exposure outside the work or home environment such as in recreational public places. Data are presented as mean ± standard deviation or number of participants with positive value for the variable (n) out of the total number of participants (N) and percentage of participants (%). Reference equations: percent predicted of normal values of spirometry, diffusing capacity, and lung volumes were calculated using Global Lung Function Initiative (GLI), Crapo, and Stock and Quanjer predicted formulas, respectively.[30-33] Percent predicted of normal values of cardiopulmonary outputs were calculated using Wassermann predicted formulas.[30] Available measures of the variables at peak exercise, at rest, and at anaerobic threshold were reported. Rate of change in the variables during the exercise testing were assessed by linear regression slope of the variables with respect to the workload. Abbreviations- BMI: body mass index; mMRC: modified medical research council; SF12: Short Form 12-Item Health Survey; IPAQ: International Physical Activity Questionnaire; CAT: COPD Assessment Test; FEV_1: forced expiratory volume in 1 second; FVC: forced vital capacity; FEF_{25-75}: maximum airflow at mid-lung volume; FEF_{75}: maximum airflow at low-lung volume; TLC: total lung capacity; RV: residual volume; FRC: functional residual capacity; D_{LCO}: single-

breath diffusing capacity of carbon monoxide; Hgb: hemoglobin; VO_2: oxygen uptake; $VO_{2Peak.kg}$: peak oxygen uptake per kilogram of body weight; Watts: work stage completed in watts; VCO_2: carbon dioxide production; V_E: minute ventilation value; RER: respiratory exchange ratio (VCO_2/VO_2) at peak exercise; RR: respiratory rate; V_T: tidal volume; HR: heart rate; HRR: heart rate reserve; SBP: systolic blood pressure; DBP: diastolic blood pressure; O_2-Pulse: oxygen uptake per heartbeat; SpO_2: oxygen saturation; VFL: volume of the tidal breath that is flow limited on expiration; EFL: expiratory flow limitation; EELV: end-expiratory lung volume; Slope of EELV across exercise stages: estimate of regression coefficient of three EELV measurements at each of baseline (rest), 20%, 40%, 60%, and 80% of the load intensity (watts) of the peak exercise stage achieved.

Table S2- Participant characteristics at baseline.

Participant characteristics	Albuterol w/ V2 Placebo w/ V3 (N=29)	Placebo w/ V2 Albuterol w/ V3 (N=20)	Overall (N=49)
**Demographics and anthropometrics**			
Age (years)	66.2±8.9	66.3±6.5	66.2±8.0
Female sex [n (%)]	25 (86.2%)	18 (90.0%)	43 (87.8%)
Height (cm)	165.5±7.8	167.9±8.6	166.5±8.1
Weight (kg)	65.3±13.5	69.1±12.0	66.9±12.9
BMI (kg/m^2)	23.7±3.5	24.5±3.9	24.0±3.7
Hemoglobin (g/dL)	13.6±0.4	13.5±0.4	13.5±0.4
**SHS Exposure**			
Ever Cabin SHS exposure [n (%)]	27 (93.1%)	14 (70.0%)	41 (83.7%)
Cabin SHS exposure among exposed (years)	16.1±9.6	20.4±7.5	17.6±9.0
Any form of non-cabin SHS exposure [n (%)]	29 (100%)	20 (100%)	49 (100%)
Childhood home SHS exposure [n (%)]	18 (62.1%)	11 (55.0%)	29 (59.2%)
Adult home SHS exposure [n (%)]	12 (41.4%)	9 (45.0%)	21 (42.9%)
Non-airline occupational SHS exposure [n (%)]	24 (82.8%)	18 (90.0%)	42 (85.7%)
Other SHS Exposure [n (%)]	26 (89.7%)	17 (85.0%)	43 (87.8%)
**Symptoms**			
mMRC Dyspnea Scale ≥1 [n (%)]	5 (31.3%)	5 (38.5%)	10 (34.5%)
SF12			
Physical component score	37.9±4.3	39.3±5.7	38.5±4.9
Mental component score	48.9±3.8	48.9±5.7	48.9±4.6
IPAQ score			
High [n (%)]	25 (86.2%)	18 (90.0%)	43 (87.8%)
Moderate [n (%)]	4 (13.8%)	1 (5.0%)	5 (10.2%)
Low [n (%)]	0 (0%)	1 (5.0%)	1 (2.0%)
CAT	6.04±5.55	7.40±5.90	6.60±5.68
Participants with any respiratory symptoms [n (%)]	28 (96.6%)	17 (85.0%)	45 (91.8%)
Participants ever experiencing shortness of breath [n (%)]	9 (31.0%)	4 (20.0%)	13 (26.5%)
Participants with cough ≥2 episodes/year [n (%)]	28 (96.6%)	16 (80.0%)	44 (89.8%)
Participants with less activity than peers [n (%)]	2 (6.9%)	2 (10.0%)	4 (8.2%)
**Pulmonary Function Tests**			
FEV_1 (% predicted)	102±18	105±9	103±16

FVC (% predicted)	106±19	110±8	107±16
FEV_1/FVC	0.75±0.05	0.75±0.04	0.75±0.05
FEV_1/FVC (% predicted)	96±6	95±6	96±6
FEF_{25-75} (% predicted)	99±33	96±26	98±30
FEF_{75} (% predicted)	127±55	127±59	127±56
D_{LCO} adjusted for Hgb (mL/min/mmHg)	20.7±4.4	22.3±4.1	21.4±4.3
D_{LCO} adjusted for Hgb (% predicted)	82±12	85 ±13	84±12
D_L/V_A adjusted for Hgb (mL/min/mmHg/L)	4.35±0.65	4.36±0.58	4.36±0.61
D_L/V_A adjusted for Hgb (% predicted)	88±13	87±10	88±12
Alveolar volume (V_A) (L)	4.87±1.02	5.12±0.99	4.97±1.00
Alveolar volume (V_A) (L) (% predicted)	91±14	95±7	93±12
TLC (% predicted)	99±12	103±11	101±11
RV (% predicted)	96±15	100±18	98±16
RV/TLC	0.39±0.07	0.38±0.05	0.39±0.07
RV/TLC (% predicted)	95±15	92±10	94±13
FRC (% predicted)	94±15	99±20	96±17
FRC/TLC	0.51±0.08	0.51±0.07	0.51±0.07
FRC/TLC (% predicted)	92±13	92±13	92±13
**Cardiopulmonary Testing Measurements**			
VO_{2Peak} (L/min)	1.27±0.40	1.42±0.43	1.33±0.41
VO_{2Peak} (% predicted)	96±22	104±18	99±21
$VO_{2Peak/kg}$ (mL/min.kg)	19.7±5.4	20.4±3.8	20.0±4.8
VO_{2Peak}/kg (% predicted)	82±17	92±24	86±20
RER_{Peak}	1.14±0.08	1.13±0.09	1.13±0.08
RER_{Peak} (% predicted)	103±7	103±8	103±8
VCO_{2Peak} (L/min)	1.45±0.49	1.62±0.52	1.52±0.50
$Watts_{Peak}$ (watts)	110±35	116±37	113±36
$Watts_{Peak}$ (% predicted)	116±28	114±29	115±28
Cumulative work (watt-minute)	503±232	582±303	535±263
Total duration (minute)	9.6±1.9	10.2±2.2	9.8±2.0
Stages completed	4.8±0.9	5.1±1.1	4.9±1.0
Symptoms at peak exercise (Borg Scale 0 to 10)			
Shortness of Breath	5.43±1.73	5.85±2.03	5.60±1.85
Effort	6.00±1.91	6.70±2.25	6.29±2.06
Fatigue	5.50±1.64	6.30±2.13	5.83±1.88
**Pulmonary Response**			
V_{EPeak} (L/min)	52.9±16.7	53.2±16.7	53.0±16.5
V_{EPeak} (% predicted)	56±12	55±13	56±12
RR_{Peak} (breaths/min)	31.4±6.1	29.4±7.4	30.6±6.7
RR_{Peak} (% predicted)	63±12	59±15	61±13
V_{TPeak} (L)	1.72±0.58	1.84±0.56	1.77±0.57
V_{TPeak} (% predicted)	94±20	99±18	96±19
V_E/VO_{2Peak}	40.9±13.5	35.7±5.9	38.8±11.3
V_E/VCO_{2Peak}	36.2±11.9	31.9±4.4	34.4±9.8

V_E/VCO_{2Peak} (% predicted)	90±30	80±11	86±24
$V_E/VCO_{2Lowest}$	33.2±12.4	29.0±4.1	31.5±10.0
$V_E/VCO_{2Lowest}$ (% predicted)	83±31	73±10	79±25
VO_2 at Anaerobic Threshold (VO_{2AT}) (L/min)	1.03±0.28	1.24±0.38	1.11±0.34
VO_2 at Anaerobic Threshold (VO_{2AT}) (% predicted)	76±23	89±21	81±23
VO_2 at Anaerobic Threshold (VO_{2AT}) (%VO_{2Peak})	84±22	88±16	86±20
V_E/VCO_2 at Anaerobic Threshold	37.8±17.2	31.4±3.6	35.2±13.7
**Cardiovascular Response**			
HR_{Rest} (beat/min)	77±14	76±26	76±19
HR_{Peak} (beat/min)	143±24	139±20	141±22
HR_{Peak} (% predicted)	93±15	90±12	92±14
HR_{Peak}≤90% predicted [n (%)]	11 (37.9%)	10 (50.0%)	21 (42.9%)
HRR	11.4±23.0	14.9±18.1	12.8±21.0
HRR≥15 [n (%)]	12 (41.4%)	11 (55.0%)	23 (46.9%)
HR_{Slope} (beat/min/10 Watts)	6.59±2.22	5.89±1.77	6.30±2.06
SBP_{Rest} (mmHg)	130±17	125±16	128±17
SBP_{Peak} (mmHg)	191±29	185±29	189±29
SBP_{Slope} (mmHg/10 Watts)	5.94±2.39	5.76±1.75	5.87±2.13
DBP_{Rest} (mmHg)	72±10	74±9	73±9
DBP_{Peak} (mmHg)	85±11	83±11	84±11
DBP_{Slope} (mmHg/10 Watts)	1.25±1.20	.0.81±0.95	1.07±1.11
O_2-$Pulse_{Rest}$ (mL/beat)	3.43±1.19	3.70±1.18	3.54±1.18
O_2-$Pulse_{Peak}$ (mL/beat)	9.32±4.15	10.4±3.21	9.74±3.80
O_2-$Pulse_{Peak}$ (% predicted)	100±29	114±31	106±30
O_2-$Pulse_{Slope}$ (mL/beat/10 Watt)	0.48±0.19	0.53±0.18	0.50±0.19
O_2-Pulse at Anaerobic Threshold (mL/beat)	8.36±2.53	10.3±3.41	9.15±3.04
O_2-Pulse at Anaerobic Threshold (% O_2-$Pulse_{Peak}$)	96±26	100±13	98±21
SpO_{2Rest}	97.7±2.1	97.3±1.5	97.5±1.8
SpO_{2Peak}	97.4±1.5	97.2±1.5	97.3±1.5
Change in SpO_2 at peak exercise	-0.28±1.56	-0.10±1.02	-0.20±1.35
**Dynamic Hyperinflation**			
VFL_{Rest} (L)	0.23±0.24	0.16±0.27	0.20±0.25
$VFL_{80\% \text{ effort}}$ (L)	0.60±0.33	0.49±0.40	0.55±0.36
VFL_{Slope} (mL/watt)	3.93±3.43	3.62±3.38	3.80±3.38
EFL_{Rest} (%)	21.9±23.6	15.8±26.4	19.5±24.7
$EFL_{80\% \text{ effort}}$ (%)	32.8±16.4	24.9±20.7	29.7±18.4
EFL_{Slope} (%/watt)	0.10±0.25	0.08±0.21	0.09±0.23
EFL at rest [n (%)]	16 (57.1%)	7 (35.0%)	23 (46.9%)
EFL at rest or during exercise [n (%)]	27 (93.1%)	16 (80.0%)	43 (87.8%)
$EELV_{Rest}$	2.68±0.47	3.04±0.80	2.82±0.64

$EELV_{80\%\ effort}$	2.68±0.49	2.87±0.72	2.76±0.59
Slope of EELV across exercise stages (mL/watts)	0.06±4.21	-1.78±2.66	-0.69±3.74
No. with increase in EELV slope [n (%)]	16 (55.2%)	5 (25.0%)	21 (42.9%)

Footnote: Demographics, secondhand smoke (SHS) exposure, symptoms, and lung function in participants with preserved spirometry that underwent exercise testing. Other SHS exposure was defined as non-aircraft cabin SHS exposure outside the work or home environment such as in recreational public places. Data are presented as mean ± standard deviation or number of participants with positive value for the variable (n) out of the total number of participants (N) and percentage of participants (%). Reference equations: percent predicted of normal values of spirometry, diffusing capacity, and lung volumes were calculated using Global Lung Function Initiative (GLI), Crapo, and Stock and Quanjer predicted formulas, respectively.[30-33] Percent predicted of normal values of cardiopulmonary outputs were calculated using Wassermann predicted formulas.[30] Available measures of the variables at peak exercise, at rest, and at anaerobic threshold were reported. Rate of change in the variables during the exercise testing were assessed by linear regression slope of the variables with respect to the workload. Abbreviations- BMI: body mass index; mMRC: modified medical research council; SF12: Short Form 12-Item Health Survey; IPAQ: International Physical Activity Questionnaire; CAT: COPD Assessment Test; FEV_1: forced expiratory volume in 1 second; FVC: forced vital capacity; FEF_{25-75}: maximum airflow at mid-lung volume; FEF_{75}: maximum airflow at low-lung volume; TLC: total lung capacity; RV: residual volume; FRC: functional residual capacity; D_{LCO}: single-breath diffusing capacity of carbon monoxide; Hgb: hemoglobin; VO_2: oxygen uptake; $VO_{2Peak.kg}$: peak oxygen uptake per kilogram of body weight; Watts: work stage completed in watts; VCO_2: carbon dioxide production; V_E: minute ventilation value; RER: respiratory exchange ratio (VCO_2/VO_2) at peak exercise; RR: respiratory rate; V_T: tidal volume; HR: heart

rate; HRR: heart rate reserve; SBP: systolic blood pressure; DBP: diastolic blood pressure; O_2-Pulse: oxygen uptake per heartbeat; SpO_2: oxygen saturation; VFL: volume of the tidal breath that is flow limited on expiration; EFL: expiratory flow limitation; EELV: end-expiratory lung volume; Slope of EELV across exercise stages: estimate of regression coefficient of three EELV measurements at each of baseline (rest), 20%, 40%, 60%, and 80% of the load intensity (watts) of the peak exercise stage achieved.

Table S3- Comparison of changes in outcomes after albuterol versus placebo treatments using paired t-test.

		Average change from baseline		
**Subgroup**	**Variable**	**Albuterol**	**Placebo**	**P value**
All completed	V_E/VCO_{2Peak}	-0.558	-2.821	0.012
	DBP_{Peak} (mmHg)	-2.2	1.6	0.048
	RV@80% (L)	-0.049	0.010	0.011
Air trapping by RV/TLC>0.35	V_{TPeak} (L/min)	0.046	-0.017	0.005
	SpO_{2Peak} (%)	0.0	-0.6	0.037
	DBP_{Peak} (mmHg)	-2.9	1.8	0.040
RV/TLC>0.35 and ≥90% adherence	V_{EPeak} (L/min)	5.107	-0.565	0.029
	O_2-$Pulse_{Peak}$ (L/min/beat)	0.600	-0.145	0.005
	VO_{2Slope} (L/min/watt)	0.001	-0.0003	0.039

Footnote: Changes in the outcomes from baseline were calculated by subtracting the subsequent visits (V2 or V3) outcome values from those of baseline visit (V1). Statistical significance was determined by a paired t-test P value of <0.05. Only outcomes with statistically significant change are shown. Abbreviations- V_E: minute ventilation value; VCO_2: carbon dioxide production; DBP: diastolic blood pressure; RV: residual volume; V_T: tidal volume; SpO_2: oxygen saturation; O_2-Pulse: oxygen uptake per heartbeat; VO_2: oxygen uptake.

SUPPLEMENTAL FIGURE

Supplemental Figure S1- Associations of Albuterol and outcomes in participants who completed the entire study. The effect of Albuterol on the outcome variables was examined with a repeated measure design using mixed-effect linear regression modeling with the random subject effect and fixed effect variables including age, sex, height, weight, and the corresponding baseline measure of the outcome variable. The number of participants (N), the summary statistics (mean±standard deviation) for each outcome variables measured in the placebo visit and the albuterol visit as well as the resulted parameter estimate (PE) representing the adjusted difference and the corresponding 95% confidence interval (CI) are shown. In this intention-to-treat analysis, N represents the number of participants who completed each (albuterol or placebo) arm of the study. The dot-and-whisker plots represent the PE and 95% CI with colors black (scaled on the top) and red (scaled on the bottom) to distinguish outcomes in which higher versus lower values are preferable. The PE and 95% CI for the statistically significant associations were shown in bold. Abbreviations: VO_2: oxygen uptake; Watts: work stage completed in watts; V_E: minute ventilation value; RR: respiratory rate; V_T: tidal volume; VCO_2: carbon dioxide production; SpO_2: oxygen saturation; O_2-Pulse: oxygen uptake per heartbeat; EELV: end-expiratory lung volume; VFL: volume of the tidal breath that is flow limited on expiration; EFL: expiratory flow limitation; SF12: Short Form 12-Item Health Survey; IPAQ: International Physical Activity Questionnaire; MET: metabolic equivalent: MVPA: moderate to vigorous physical activities; PE: parameter estimate; CI: confidence interval.

Outcome	Placebo (N=42)	Albuterol (N=42)	PE (95% CI)
	Mean±Standard Deviation		*Adjusted for baseline and covariates*
**Peak exercise**			
VO_2 (L/min)	1.40±0.39	1.41±0.39	0.009 (−0.045, 0.063)
Watts (watts)	117.3±36.6	116.1±33.3	−1.190 (−4.742, 2.361)
V_E (L/min).y	55.05±14.87	57.41±15.59	2.353 (−0.192, 4.899)
RR (breaths/min)	33±8	33±8	0.659 (−0.902, 2.220)
V_T (L)	1.72±0.48	1.75±0.46	0.037 (−0.001, 0.074)
V_E/VO_2	37.8±6.2	39.4±7.2	**1.581 (0.015, 3.146)**
V_E/VCO_2	32.0±4.4	34.3±6.7	**2.262 (0.588, 3.936)**
SpO_2	97±2	97±3	0.249 (−0.240, 0.739)
Heart rate (beat/min)	145±17	146±18	0.929 (−3.325, 5.182)
O_2-Pulse (mL/beat)	9.6±2.5	9.6±2.3	0.002 (−0.451, 0.456)
Systolic blood pressure (mmHg)	189±23	185±24	−3.293 (−9.245, 2.660)
Diastolic blood pressure (mmHg)	84±10	81±12	**−3.844 (−7.423, −0.265)**
Borg scale - Shortness of breath	6.0±1.7	5.9±1.9	−0.146 (−0.632, 0.339)
Borg scale - Fatigue	6.3±1.6	6.0±1.8	−0.268 (−0.751, 0.215)
Borg scale - Effort	6.8±2.1	6.4±2.1	−0.366 (−0.992, 0.260)
**Anaerobic threshold**			
VO_2 (L/min)	1.18±0.37	1.17±0.35	−0.012 (−0.087, 0.063)
Heart rate (beat/min)	120±15	121±15	0.857 (−2.760, 4.475)
O_2-Pulse (mL/beat)	10.0±2.9	9.6±2.5	−0.244 (−0.804, 0.317)
**Dynamic hyperinflation**			
EELV slope (mL/watt)	−0.8±3.4	−0.7±3.2	0.122 (−0.678, 0.922)
VFL at 80% effort (L)	0.57±0.37	0.6±0.4	0.008 (−0.068, 0.085)
VFL slope (mL/watt)	3.7±3.9	−3.6±3.9	−0.106 (−1.031, 0.820)
EFL at 80% effort (%)	32±22	32±22	−0.483 (−3.691, 2.725)
EFL slope (%/watt)	0.05±0.28	0.05±0.25	−0.001 (−0.057, 0.054)
V_T slope (mL/watt)	10.4±5.3	10.3±5.1	−0.075 (−0.995, 0.845)
**Symptoms at rest**			
mMRC score	0.2±0.4	0.2±0.4	−0.067 (−0.290, 0.157)
SF12 - Physical component score	39.4±4.5	38.5±4.8	−1.059 (−2.617, 0.498)
SF12 - Mental component score	48.8±3.6	49.5±4.1	0.731 (−0.540, 2.001)
**Daily physical activity**			
IPAQ - Total physical MET (kcal/kg/hour)	19.63±14.88	18.85±15.70	−0.774 (−4.811, 3.262)
Total MVPA (1000 min)	0.05±0.04	0.05±0.04	0.005 (−0.007, 0.017)
Percent of time in MVPA (%)	10.9±8.7	11.6±7.8	0.914 (−1.588, 3.416)
Highest METs (kcal/kg/hour)	1.39±0.33	1.40±0.27	0.013 (−0.079, 0.104)

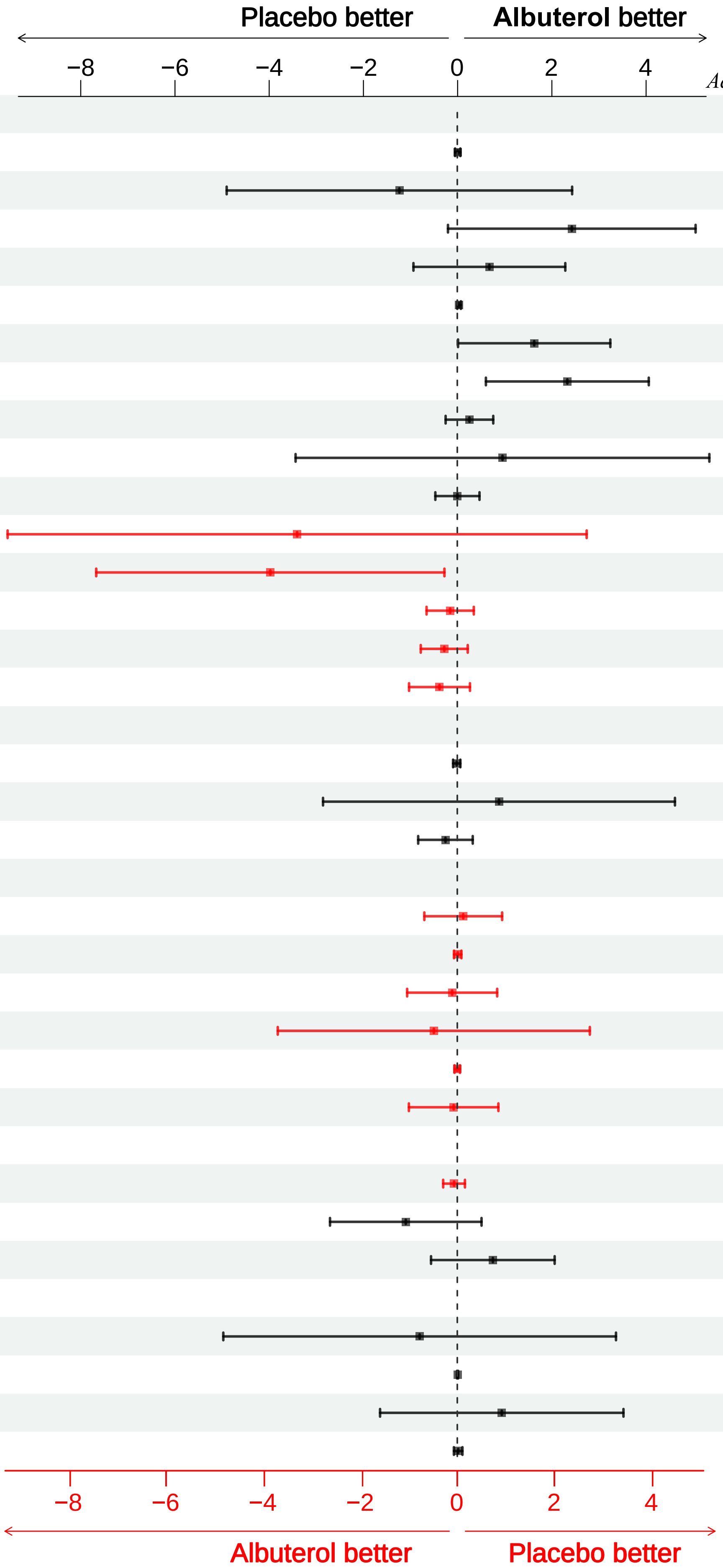

REFERENCES

References

1. Eisner MD, Wang Y, Haight TJ, Balmes J, Hammond SK, Tager IB. Secondhand smoke exposure, pulmonary function, and cardiovascular mortality. *Ann Epidemiol* 2007; **17**(5): 364-73.
2. Arjomandi M, Haight T, Redberg R, Gold WM. Pulmonary function abnormalities in never-smoking flight attendants exposed to secondhand tobacco smoke in the aircraft cabin. *J Occup Environ Med* 2009; **51**(6): 639-46.
3. Arjomandi M, Haight T, Sadeghi N, Redberg R, Gold WM. Reduced exercise tolerance and pulmonary capillary recruitment with remote secondhand smoke exposure. *PLoS One* 2012; **7**(4): e34393.
4. Koeverden I, Blanc PD, Bowler RP, Arjomandi M. Secondhand Tobacco Smoke and COPD Risk in Smokers: A COPDGene Study Cohort Subgroup Analysis. *COPD* 2015; **12**(2): 182-9.
5. Comroe Jr. JH. Pulmonary Function Tests. Methods in Medical Research. Chicago, Illinois: Year Book Publishers, Inc.; 1950: 188.
6. Mitchell MM, Renzetti AD, Jr. Evaluation of a single-breath method of measuring total lung capacity. *Am Rev Respir Dis* 1968; **97**(4): 571-80.
7. Burns CB, Scheinhorn DJ. Evaluation of single-breath helium dilution total lung capacity in obstructive lung disease. *Am Rev Respir Dis* 1984; **130**(4): 580-3.
8. Dubois AB, Botelho SY, Bedell GN, Marshall R, Comroe JH, Jr. A rapid plethysmographic method for measuring thoracic gas volume: a comparison with a nitrogen washout method for measuring functional residual capacity in normal subjects. *J Clin Invest* 1956; **35**(3): 322-6.
9. Dubois AB, Botelho SY, Comroe JH, Jr. A new method for measuring airway resistance in man using a body plethysmograph: values in normal subjects and in patients with respiratory disease. *J Clin Invest* 1956; **35**(3): 327-35.
10. Briscoe WA, Dubois AB. The relationship between airway resistance, airway conductance and lung volume in subjects of different age and body size. *J Clin Invest* 1958; **37**(9): 1279-85.
11. Blakemore WS, Forster RE, Morton JW, Ogilvie CM. A standardized breath holding technique for the clinical measurement of the diffusing capacity of the lung for carbon monoxide. *J Clin Invest* 1957; **36**(1 Part 1): 1-17.
12. Standardization of Spirometry, 1994 Update. American Thoracic Society. *Am J Respir Crit Care Med* 1995; **152**(3): 1107-36.
13. Macintyre N, Crapo RO, Viegi G, et al. Standardisation of the single-breath determination of carbon monoxide uptake in the lung. *Eur Respir J* 2005; **26**(4): 720-35.
14. Miller MR, Crapo R, Hankinson J, et al. General considerations for lung function testing. *Eur Respir J* 2005; **26**(1): 153-61.
15. Miller MR, Hankinson J, Brusasco V, et al. Standardisation of spirometry. *Eur Respir J* 2005; **26**(2): 319-38.
16. Pellegrino R, Viegi G, Brusasco V, et al. Interpretative strategies for lung function tests. *Eur Respir J* 2005; **26**(5): 948-68.
17. Wanger J, Clausen JL, Coates A, et al. Standardisation of the measurement of lung volumes. *Eur Respir J* 2005; **26**(3): 511-22.
18. Beaver WL, Wasserman K, Whipp BJ. A new method for detecting anaerobic threshold by gas exchange. *J Appl Physiol* 1986; **60**(6): 2020-7.

19. Wasserman K, Hansen JE, Sue DY, BJ W. Principles of Exercise Testing and Interpretation. Philadelphia: Lea & Febiger; 1987.
20. O'Donnell DE, Lam M, Webb KA. Measurement of symptoms, lung hyperinflation, and endurance during exercise in chronic obstructive pulmonary disease. *American journal of respiratory and critical care medicine* 1998; **158**(5 Pt 1): 1557-65.
21. O'Donnell DE, Revill SM, Webb KA. Dynamic hyperinflation and exercise intolerance in chronic obstructive pulmonary disease. *American journal of respiratory and critical care medicine* 2001; **164**(5): 770-7.
22. Chen J, Weldemichael L, Zeng S, et al. Actigraphy informs distinct patient-centered outcomes in Pre-COPD. *Respir Med* 2021; **187**: 106543.
23. Trost SG, McIver KL, Pate RR. Conducting accelerometer-based activity assessments in field-based research. *Med Sci Sports Exerc* 2005; **37**(11 Suppl): S531-43.
24. Jones PW, Harding G, Berry P, Wiklund I, Chen WH, Kline Leidy N. Development and first validation of the COPD Assessment Test. *Eur Respir J* 2009; **34**(3): 648-54.
25. Fletcher CM, Elmes PC, Fairbairn AS, Wood CH. The significance of respiratory symptoms and the diagnosis of chronic bronchitis in a working population. *Br Med J* 1959; **2**(5147): 257-66.
26. Jenkinson C, Layte R. Development and testing of the UK SF-12 (short form health survey). *J Health Serv Res Policy* 1997; **2**(1): 14-8.
27. Craig CL, Marshall AL, Sjöström M, et al. International physical activity questionnaire: 12-country reliability and validity. *Med Sci Sports Exerc* 2003; **35**(8): 1381-95.
28. Hajiro T, Nishimura K, Jones PW, et al. A novel, short, and simple questionnaire to measure health-related quality of life in patients with chronic obstructive pulmonary disease. *Am J Respir Crit Care Med* 1999; **159**(6): 1874-8.
29. Borg G. Borg's perceived exertion and pain scales. Champaign, IL, US: Human Kinetics; 1998.
30. Wasserman K, Hansen J, Sue D, Stringer W, Whipp B. Principles of Exercise Testing and Interpretation. 4th edition ed: Lippincott Williams & Wilkins, Philadelphia, USA.; 2004.
31. Stocks J, Quanjer PH. Reference values for residual volume, functional residual capacity and total lung capacity. ATS Workshop on Lung Volume Measurements. Official Statement of The European Respiratory Society. *Eur Respir J* 1995; **8**(3): 492-506.
32. Quanjer PH, Stanojevic S, Cole TJ, et al. Multi-ethnic reference values for spirometry for the 3-95-yr age range: the global lung function 2012 equations. *Eur Respir J* 2012; **40**(6): 1324-43.
33. Crapo RO, Morris AH, Gardner RM. Reference values for pulmonary tissue volume, membrane diffusing capacity, and pulmonary capillary blood volume. *Bull Eur Physiopathol Respir* 1982; **18**(6): 893-9.
